# Supplementary material for: Long-term monitoring of ultratrace nucleic acids using tetrahedral nanostructure-based NgAgo on wearable microneedles
Source: Nat Commun. 2024 Mar 2;15:1936. doi: 10.1038/s41467-024-46215-w (PMC10908814; doi:10.1038/s41467-024-46215-w)
Supplement: Supplementary file 1 — Supplementary Information [file 41467_2024_46215_MOESM1_ESM.pdf]

Long-Term Monitoring of Ultratrace Nucleic Acids using Tetrahedral Nanostructure-Based NgAgo on Wearable Microneedles

Bin Yang, Haonan Wang, Jilie Kong, Xueen Fang*

Department of Chemistry and Institutes of Biomedical Sciences, Fudan University, Shanghai, 200433, PR China

Corresponding author emails:

fxech@fudan.edu.cn (X. E. Fang)

Content

Supplementary Figure 1. Hydrophobic testing of SU-8 microneedle patch.

Supplementary Figure 2. Fabrication schematic of SU-8 MN patch.

Supplementary Figure 3. Optical photograph of the three-in-one SU-8 microneedle patch.

Supplementary Figure 4. Mechanical and electrical properties of the SU-8 MN patch.

Supplementary Figure 5. Illustration of the TPU patch.

Supplementary Figure 6. The optimization of TPU proportion in DMF during fabrication.

Supplementary Figure 7. Electrical performance of the functionalized TPU film under different distortion.

Supplementary Figure 8. The prepared TPU film had a good flexibility and stretchability under deformation.

Supplementary Figure 9. Raw data of the functionalized TPU film via a stylus profiler.

Supplementary Figure 10. Viability of CNE cells after treatment with the different materials, respectively for 28 h.

Supplementary Figure 11. Verification of TDN-Ng biosensing interface.

Supplementary Figure 12. The relationship between interface modification and signal response based on double-stranded DNA ladder from 10 bp to 70 bp.

Supplementary Figure 13. Characterization of different TDNs.

Supplementary Figure 14. AFM phase photograph of TDN-17/NgAgo protein.

Supplementary Figure 15. Schematic of the TDN modified bio-interfaces for investigation on the impact of different TDN modified biosensing interfaces.

Supplementary Figure 16. Illustration of Gaussian Box.

Supplementary Figure 17. Characterization of the engineered NgAgo protein.

Supplementary Figure 18. Optimization and screening for guide DNA targeting to target DNA.

Supplementary Figure 19. CV plots of four gDNA-guided Ng system for recognizing target DNA, using 0.05 M $[Fe(CN)_6]^{3-/4-}$.

Supplementary Figure 20. The illustration of SPR method.

Supplementary Figure 21. Molecular dynamic simulations of NgAgo protein and guide DNA.

Supplementary Figure 22. The graphene surface with Ng system for recognizing target EBV DNA, in the range of 0, 3×10^{-12} M, 3×10^{-11} M, 3×10^{-10} M, 6×10^{-10} M, using 0.05 M $[Fe(CN)_6]^{3-/4-}$.

Supplementary Figure 23. The gDNA3-guided Ng system without graphene surface for recognizing target EBV DNA.

Supplementary Figure 24. The investigation on the gDNA-guided Ng system binding with target DNA validated by UV-vis spectrum.

Supplementary Figure 25. The comparison of gDNA3-guided Ng system and g3 linker for recognizing target EBV DNA, using 0.05 M $[Fe(CN)_6]^{3-/4-}$.

Supplementary Figure 26. End-point quantitative detection of EBV cfDNA by graphene-TDN-Ng platform.

Supplementary Figure 27. Stable sensitivity tests *in vitro*.

Supplementary Figure 28. RT-PCR primers screening for RNA-168

Supplementary Figure 29. RT-PCR primers screening for RNA-351.

Supplementary Figure 30. Dynamic curves and calibration of RNA-168 and RNA-351 by RT-PCR.

Supplementary Figure 31. Genotyping of RNA-168 and RNA-351 extracted from CNE-Luc cell lines.

Supplementary Figure 32. Gene-typing of different sequences.

Supplementary Figure 33. ISF sampling method.

Supplementary Figure 34. Paralleled demonstrations of mice for RNA biomarkers monitoring.

Supplementary Figure 35. Stable sensitivity tests *in vivo*.

Supplementary Figure 36. Demonstration of the integrated wearable electronics on immunodeficiency mouse.

Supplementary Figure 37. Dynamic curves and calibration of SA and PA by PCR.

Supplementary Figure 38. Raw data of continuous parallel demonstrations on sepsis mice bearing SA and PA strain at different time points for 36 h.

Supplementary Figure 39. Paralleled demonstrations on sepsis mice bearing SA and PA strain at different time points within 36 h recorded by gold standard PCR.

Figure 40. Selectivity of TDN-Ng platform for different cell lines lysate.

Supplementary Figure 41. The application of TDN-Ng sensor for real-time monitoring SA and PA target DNA in vitro, PBS (0.01 M, pH 7.4), 37°C, reverse iontophoresis of 10 V.

Supplementary Figure 42. Specificity in vivo detection of the TDN-Ng MN patch for three different animal models, at the 4h time point.

Supplementary Figure 43. Selectivity of the sensor with scrambled guide DNA under different nucleic acid of 0.3 nM in vitro.

Supplementary Figure 44. Electrophoretic mobility shift assay (EMSA) of Ng system.

Supplementary Figure 45. Characterization of the MN patch.

Supplementary Figure 46. Characterization of the TPU patch.

Supplementary Figure 47. Mechanical performance of the TPU patch.

Supplementary Figure 48. Surface characterization of the printed TPU patch.

Supplementary Note 1. Comparison on different Ng/gDNA modified graphene bio-interfaces based on double-stranded DNA ladder.

Supplementary Note 2. Real-time monitoring ability of TDN-Ng bio-interfaced microelectrode.

Supplementary Note 3. Theory and deduction of Gaussian Box based on Gouy-Chapman-Stern model.

Supplementary Note 4. RMSD for NgAgo protein and guide DNA during molecular dynamic simulations.

Supplementary Note 5. Investigation on the binding ability of gDNA3-guided Ng system.

Supplementary Note 6. Theory on the TDN-Ng system reaction rate.

Supplementary Note 7. Discussion on TDN-Ng interface for recognizing target DNA by end-point method.

Supplementary Note 8. Primer screening, calibration, gene-typing for EBV RNA

Supplementary Method 1. Fabrication of the biosensor based on dsDNA ladder-Ng system

Supplementary Method 2. Fabrication of the biosensor based on Ng system for recognizing cfDNA and RNA

Supplementary Table 1 Nucleic acid sequences in the experiments.

Supplementary Table 2. Comparison of representative amplification-free strategy.

Supplementary Table 3. The comparison of state-of-the-art MN patches for diagnostics.

Supplementary Reference

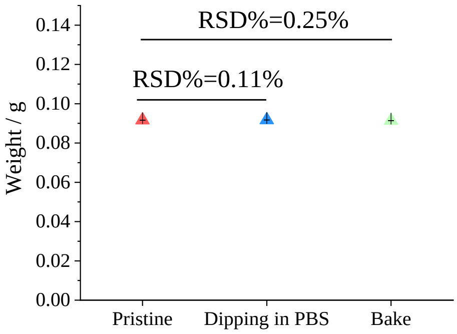

Supplementary Figure 1. **Hydrophobic testing of SU-8 microneedle patch.** The microneedles were firstly immersed in PBS (pH 7.4, 37 ° C), and then put in the oven (37 ° C, 20 min). Examine its quality changes in different conditions. As shown, compared with the original SU-8 microneedle (Pristine group in blue), there was little changes in PBS (pink) and after drying (dark yellow) and RSD% were 0.11% and 0.25%, respectively. These results preliminarily indicated that the SU-8 microneedle has certain hydrophobicity and structural stability.

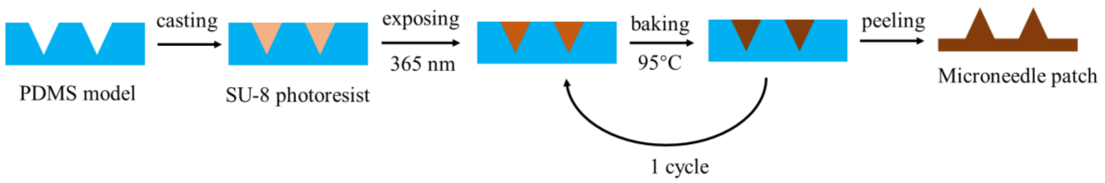

Supplementary Figure 2. **Fabrication schematic of SU-8 MN patch**. The processes include casting, exposing, baking, peeling, the detailed was listed in Method section.

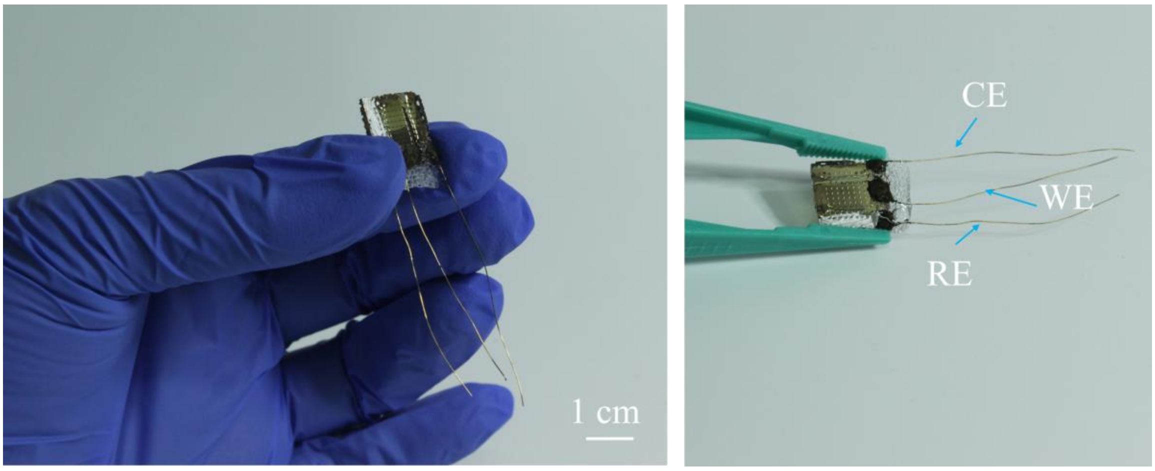

Supplementary Figure 3. **Optical photograph of the three-in-one SU-8 microneedle patch**. CE, WE, RE referring to counter electrode, working electrode, reference electrode.

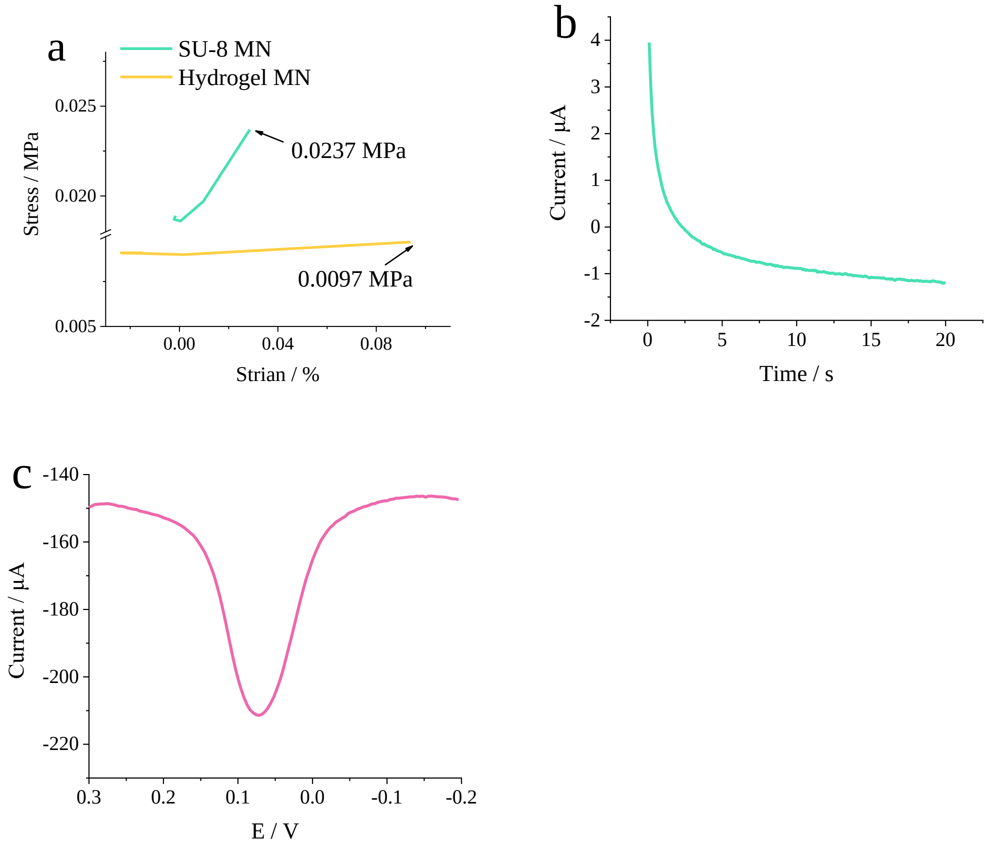

Supplementary Figure 4. **Mechanical and electrical properties of the SU-8 MN patch.** (a) Compressing test of two different MN patch. (b) I-T curve of the three-in-on SU-8 MN, using PBS buffer, pH 7.4, 0.01 M. (c) DPV curve of the three-in-one SU-8 MN, using 0.05 M $[Fe(CN)_6]^{3-/4-}$.

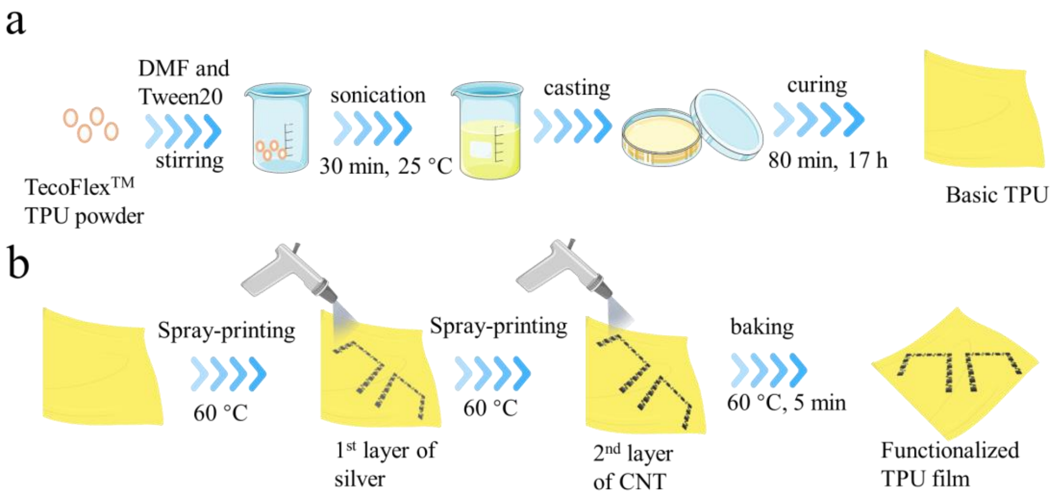

Supplementary Figure 5. **Illustration of the TPU patch.** Including basic TPU (a) and functionalized TPU with conductive patterns (b).

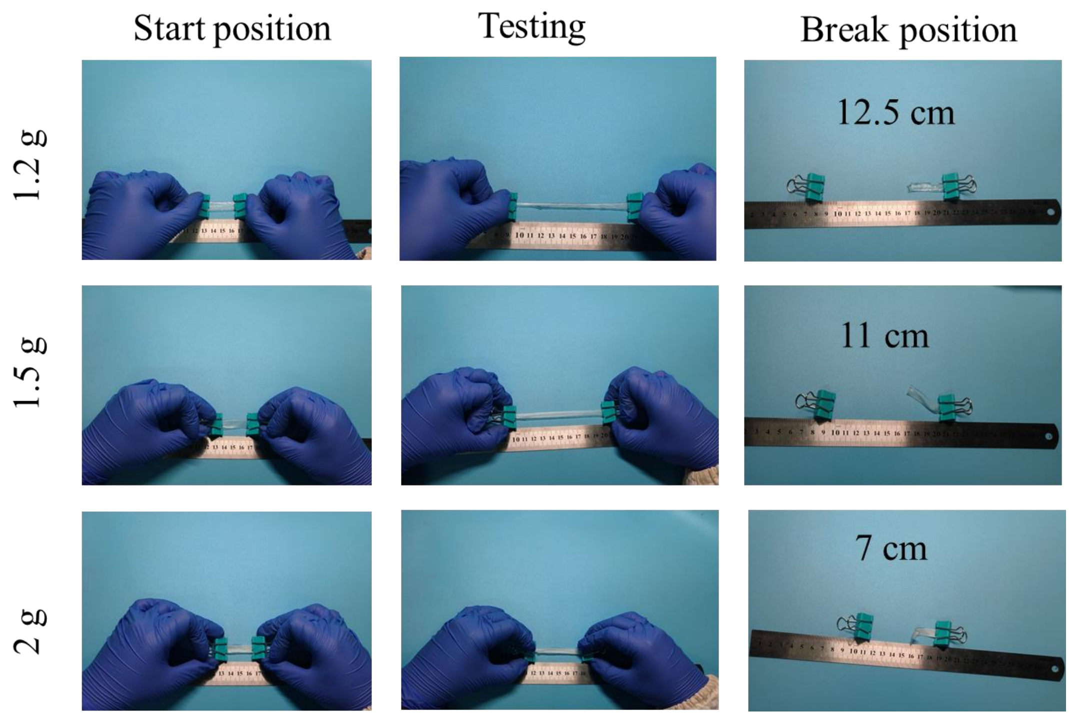

Supplementary Figure 6. **The optimization of TPU proportion in DMF during fabrication.** We can see that the elongation decreased as the content of TPU powder increased. Finally, the proportion of 1.2 g TPU powder was chosen for the subsequence experiments.

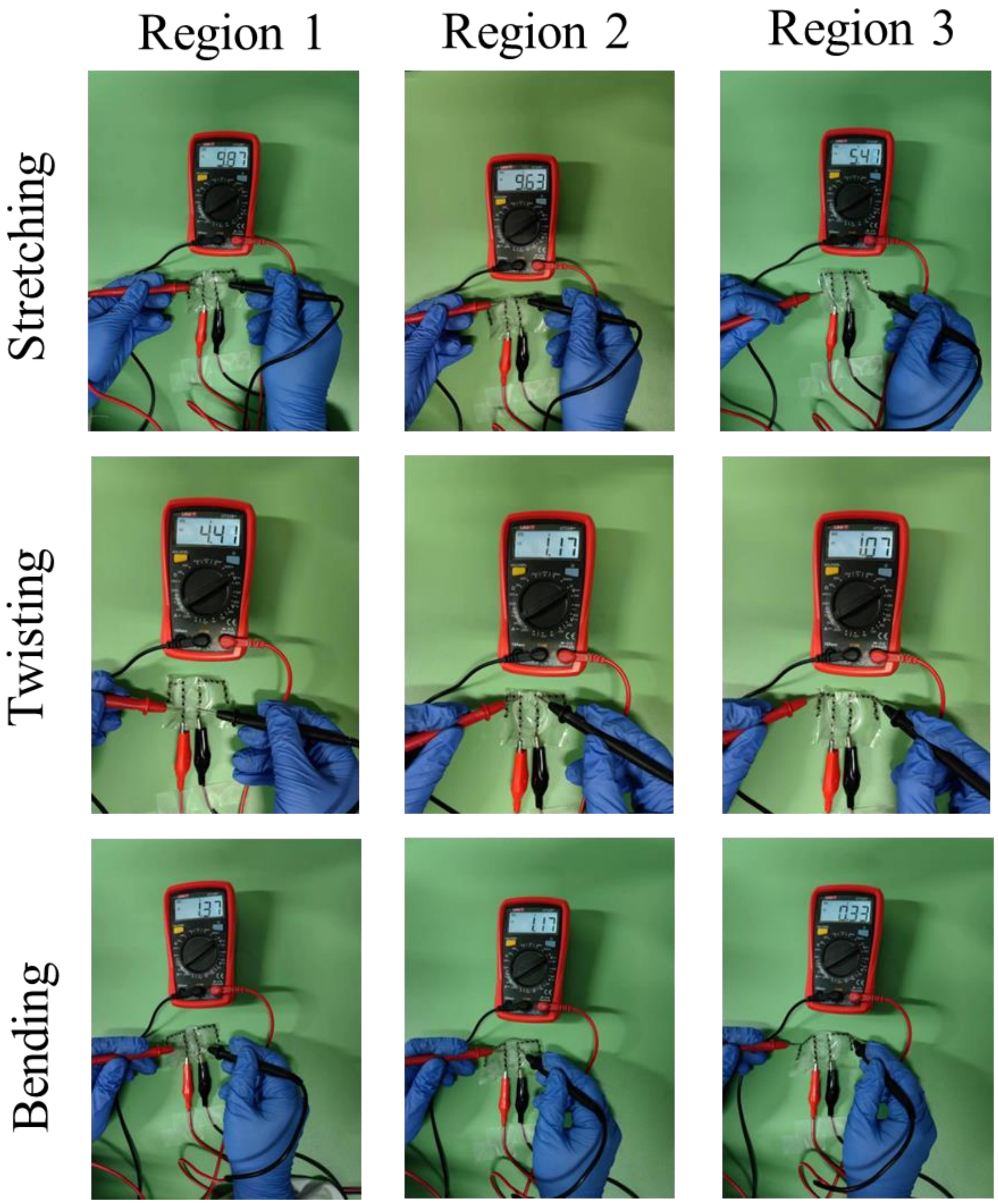

Supplementary Figure 7. **Electrical performance of the functionalized TPU film under different distortion.** With 10 V outer direct voltage, we can see that the TPU had good electrical properties within its conductive pattern.

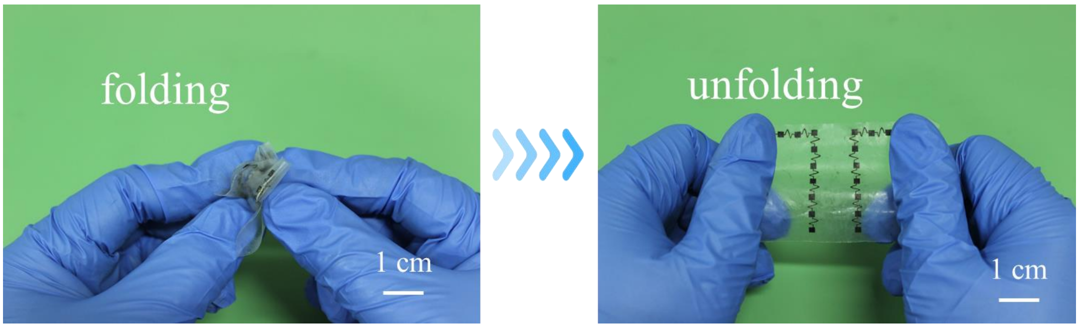

Supplementary Figure 8. **The prepared TPU film had a good flexibility and stretchability under deformation**.

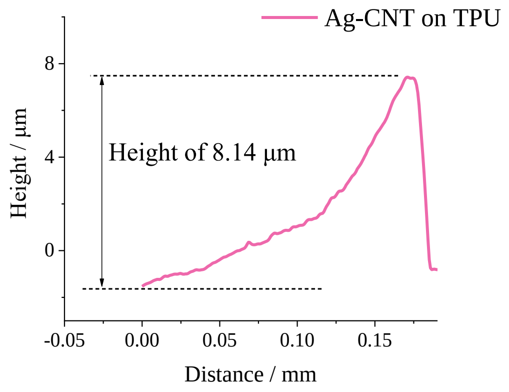

Supplementary Figure 9. **Raw data of the functionalized TPU film via a stylus profiler.**

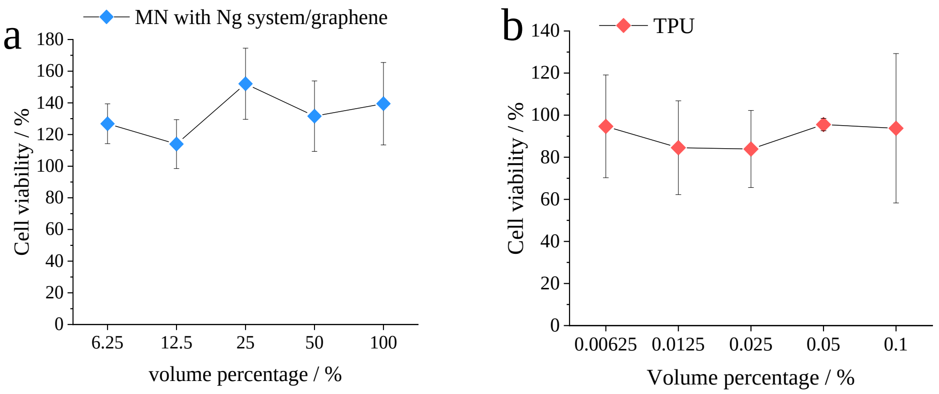

Supplementary Figure 10. **Viability of CNE cells after treatment with different materials, respectively for 28 h.** (a) Incubation with microneedle/Ng system/graphene. (b) Incubation with TPU material. These two tests were according to an MTT assay with PBS as a blank (n=5 independent experiments).

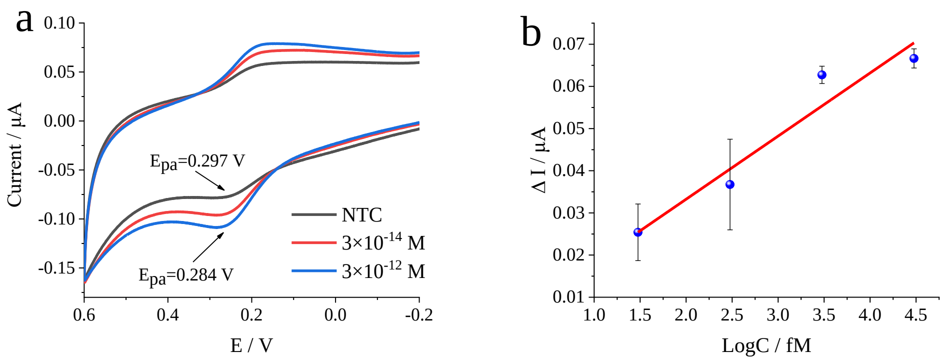

Supplementary Figure 11. **Verification of TDN-Ng biosensing interface.** (a) CV plot for the as-prepared TDN-Ng micro-electrode under different conditions, scanning rate of 0.05 V/s, sampling interval of 0.001 V. (b) Calibration curves for different concentration of target DNA. All the data was analyzed by Origin software.

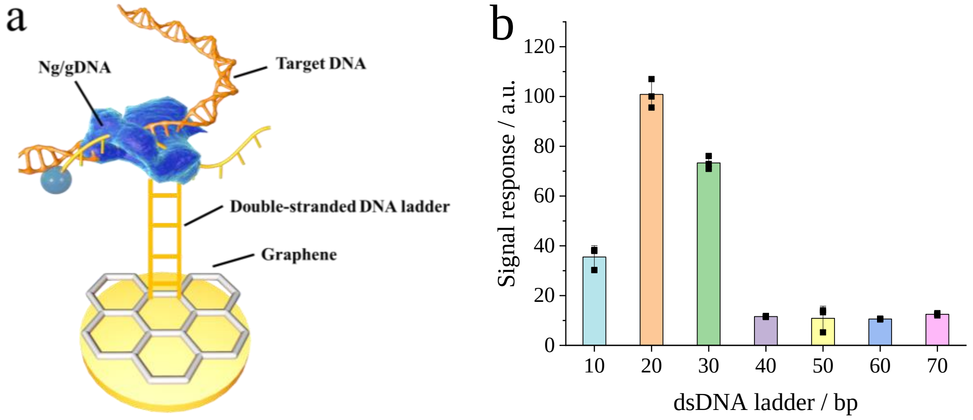

Supplementary Figure 12. **The relationship between interface modification and signal response based on double-stranded DNA ladder from 10 bp to 70 bp.** (a) Schematic of the DNA ladder interfaces, from 3 nm to 21 nm. (b) The signal response of different DNA ladders in simulated ISF (PBS, pH=7.4, 37°C, 0.01M).

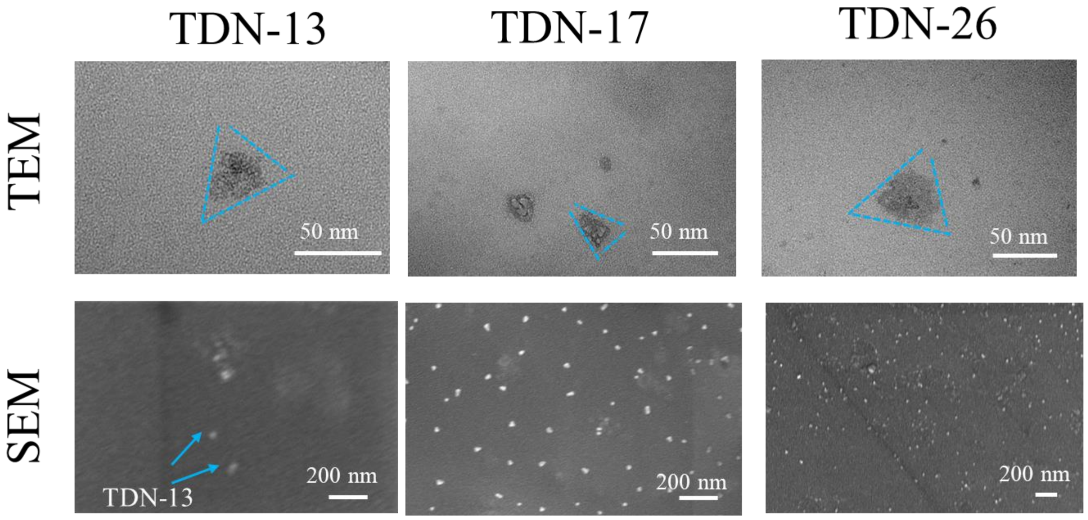

Supplementary Figure 13. **Characterization of TDNs.** Morphology characterization of TDN-13, TDN-17, TDN-26 by TEM and SEM.

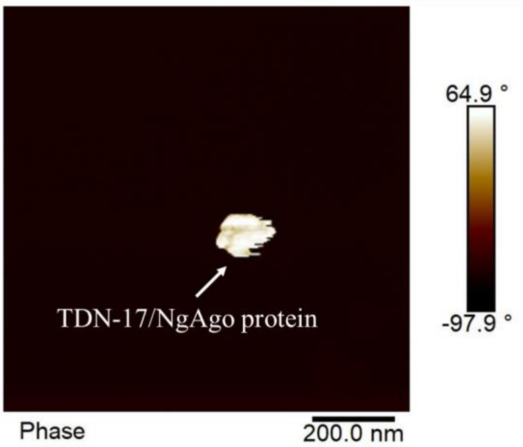

Supplementary Figure 14. **AFM phase image of TDN-17/NgAgo protein.** Scale bar: 200 nm.

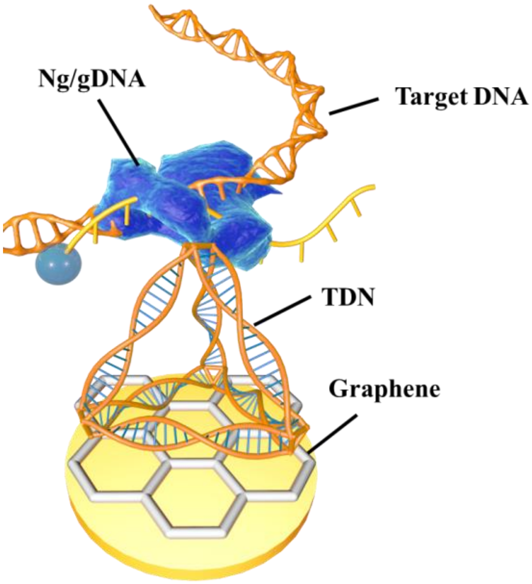

Supplementary Figure 15. **Schematic of the TDN modified bio-interfaces for investigation on the impact of different TDN modified biosensing interfaces.**

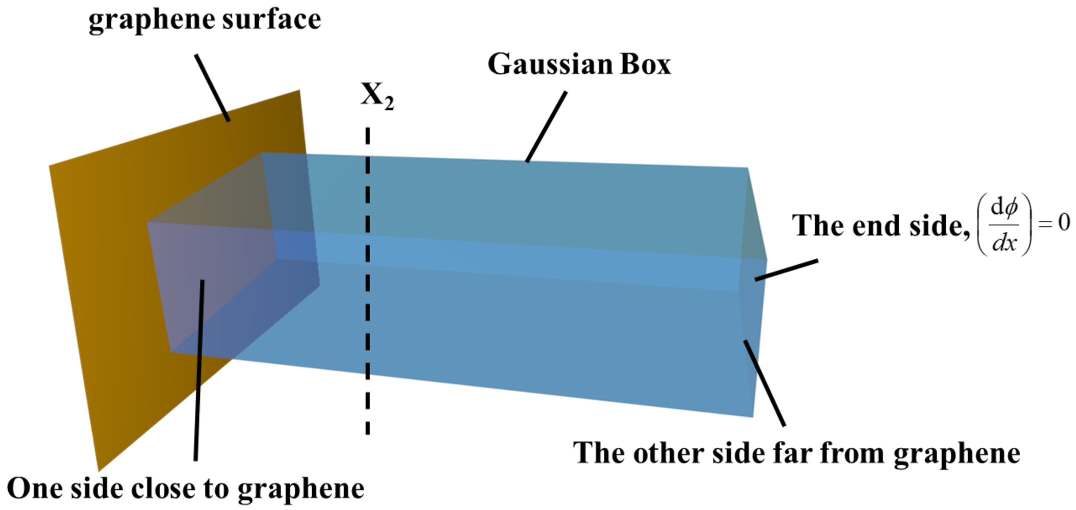

Supplementary Figure 16. **Illustration of Gaussian Box.**

a

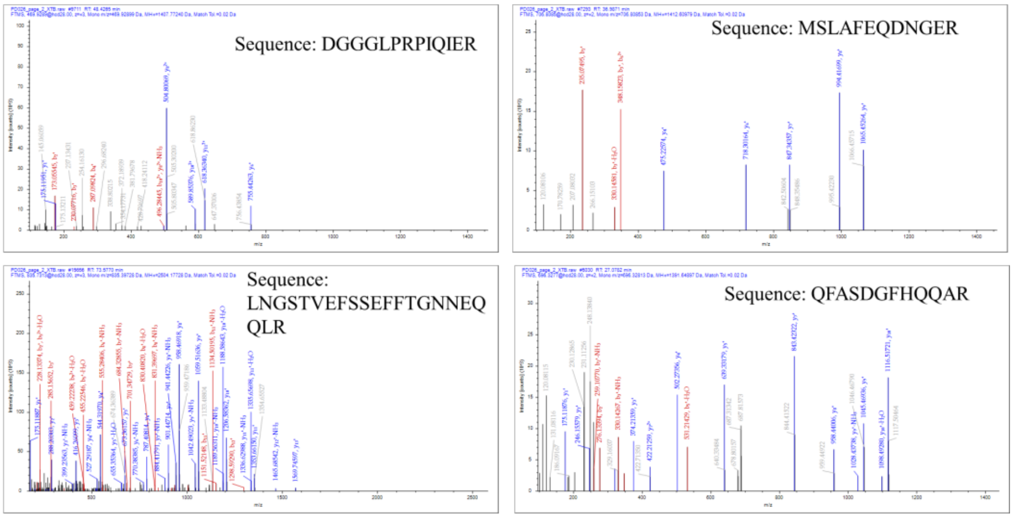

b

Protein FD	Master	Accession	Description	Exp. q-val	Contamin	Sum PEP S	Coverage	# Peptides	# PSMs	# Unique I	# AAs	MW [kDa]	calc. pI	Score Seq	# Peptides	# Protein (
High	Master Protein	L0AJX6	Stem cell self-renewal protein Piwi domain protein OS=Natronobacterium gregoryi (	0	FALSE	105.933	31	24	108	24	887	98.2	4.73	200.58	24	1
High	Master Protein	A0A238YGX6	DUF1902 domain-containing protein OS=Halorubrum vacuolatum OX=63740 GN=SA	0	FALSE	1.621	8	1	2	1	99	11.5	4.77	0	1	1
High	Master Protein	A0A238VAA4	Ribokinase OS=Halorubrum vacuolatum OX=63740 GN=SAMN06264855_102127 PE	0	FALSE	1.242	6	1	1	1	382	39.2	4.34	0	1	1
High	Master Protein	D3T2D6	Virus protein phiCh1-VP84 OS=Natrialba magadii (strain ATCC 43099 / DSM 3394 / C	0	FALSE	1.208	3	1	1	1	254	29.8	4.55	0	1	1
High	Master Protein	A0A1H1AQ48	Probable tRNA pseudouridine synthase D OS=Natronobacterium texcoconense OX=	0	FALSE	1.097	9	1	1	1	450	49.9	4.6	0	1	1
High	Master Protein	A0A238XN68	Alpha-galactosidase OS=Halorubrum vacuolatum OX=63740 GN=SAMN06264855_1	0	FALSE	1.054	2	1	1	1	667	72.8	4.59	0	1	1
High	Master Protein	D3SUJ1	DEAD/DEAH box helicase OS=Natrialba magadii (strain ATCC 43099 / DSM 3394 / C(	0	FALSE	1.009	2	1	2	1	785	86.1	4.67	1.75	1	1
High	Master Protein	A0A1I3J2Q4	PAS domain S-box-containing protein OS=Natronobacterium gregoryi OX=44930 GN	0	FALSE	0.997	2	1	2	1	637	70.6	4.45	0	1	1
High	Master Protein	L0ALW1	Lycopene cyclase domain protein OS=Natronobacterium gregoryi (strain ATCC 4309	0	FALSE	0.982	3	1	6	1	263	29.3	6.93	0	1	1
High	Master Protein	A0A1I3T3N8	Cytochrome c oxidase assembly protein subunit 15 OS=Natronobacterium gregoryi	0.009	FALSE	0.957	2	1	1	1	284	30.3	9.5	0	1	1
High	Master Protein	A0A1H1A979	Polyketide cyclase / dehydrase and lipid transport OS=Natronobacterium texcocone	0.009	FALSE	0.947	4	1	1	1	181	20.8	4.88	0	1	1
Medium	Master Protein	A0A238WLP2	Glycerol-1-phosphate dehydrogenase [NAD(P)+] OS=Halorubrum vacuolatum OX=6	0.018	FALSE	0.92	14	1	1	1	348	36.6	4.83	0	1	1
Medium	Master Protein	A0A1H1IQQ1	BFN domain-containing protein OS=Natronobacterium texcoconense OX=1095778 (	0.018	FALSE	0.871	6	1	1	1	155	16.8	4.25	0	1	1
Medium	Master Protein	L9YJ76	Transposase (Fragment) OS=Natronobacterium gregoryi (strain ATCC 43098 / DSM	0.018	FALSE	0.856	5	1	2	1	134	14.7	6.79	0	1	1
Medium	Master Protein	A0A1H0YWK6	Creatinine amidohydrolase OS=Natronobacterium texcoconense OX=1095778 GN=S	0.026	FALSE	0.823	13	1	1	1	245	26.3	4.63	0	1	1

Supplementary Figure 17. **Characterization of the engineered NgAgo protein.** (a) Secondary mass spectrometry of partial unique peptide. (b) Analysis results predicted from the mass spectrometry.

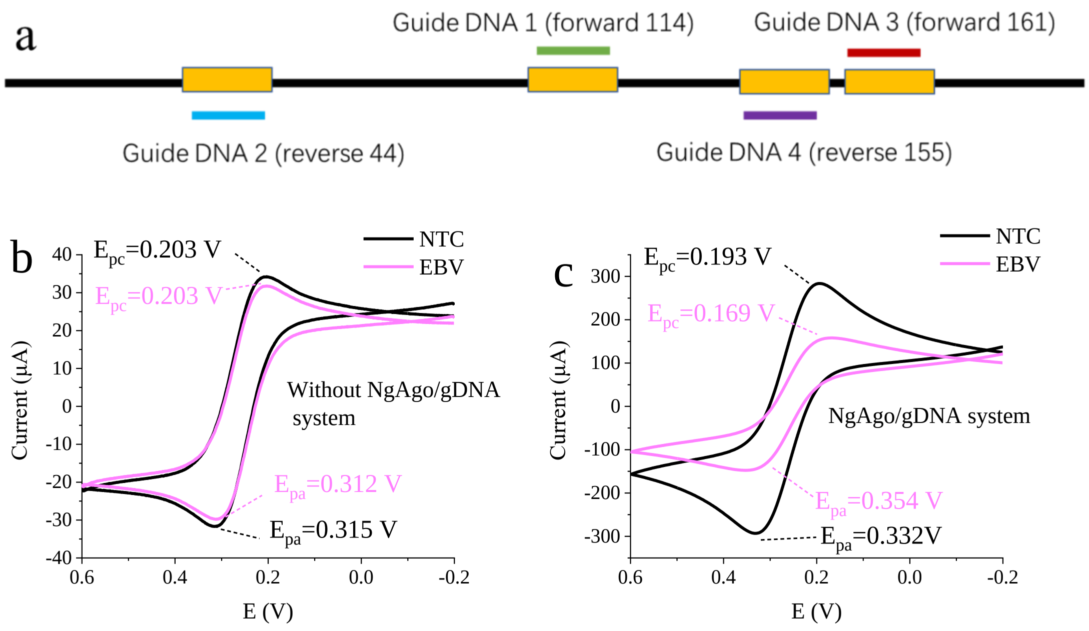

Supplementary Figure 18. **Optimization and screening for guide DNA targeting to target DNA.** (a) Schematic of guide DNA (gDNA) screening and design. (b) CV plots of the graphene surface without Ng system, using 0.005 M $[Fe(CN)_6]^{3-/4-}$. (c) CV plots of the graphene surface with Ng system, using 0.05 M $[Fe(CN)_6]^{3-/4-}$.

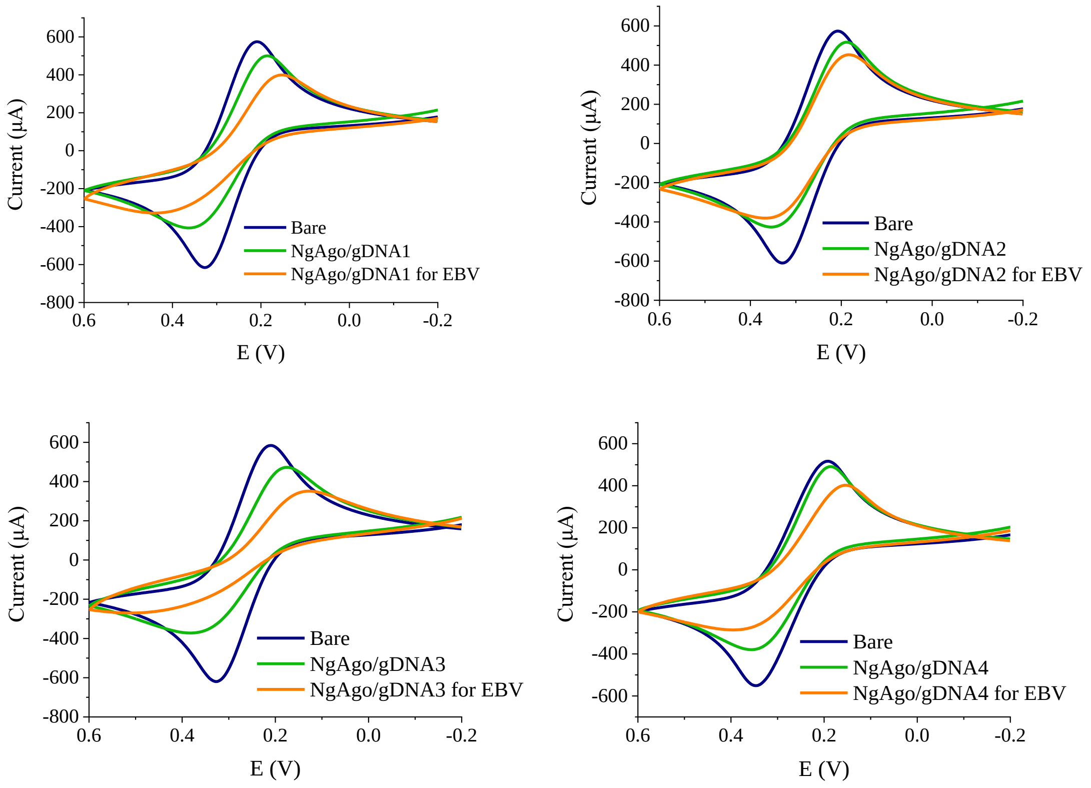

Supplementary Figure 19. **CV plots of four gDNA-guided Ng system for recognizing target DNA, using 0.05 M $[Fe(CN)_6]^{3-/4-}$.** After the screening of different guide DNA, guide DNA 3 was chosen as the optimal guide sequence.

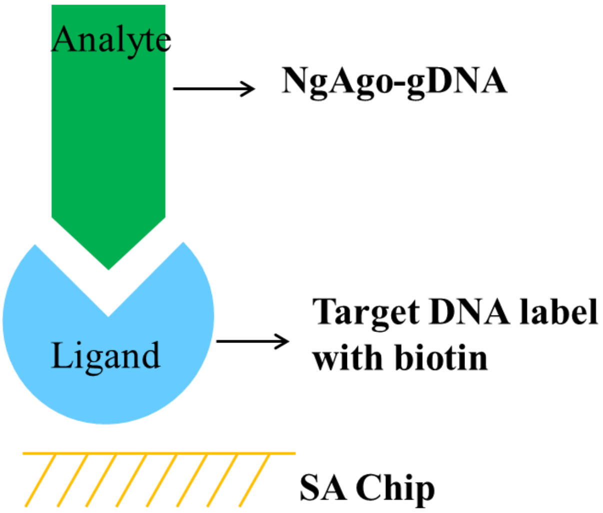

Supplementary Figure 20. **The illustration of SPR method.** Firstly, target DNA as the ligand was immobilized on the surface of the chip, then the analyte of NgAgo-gDNA was flowed into the channel to combine with the ligand.

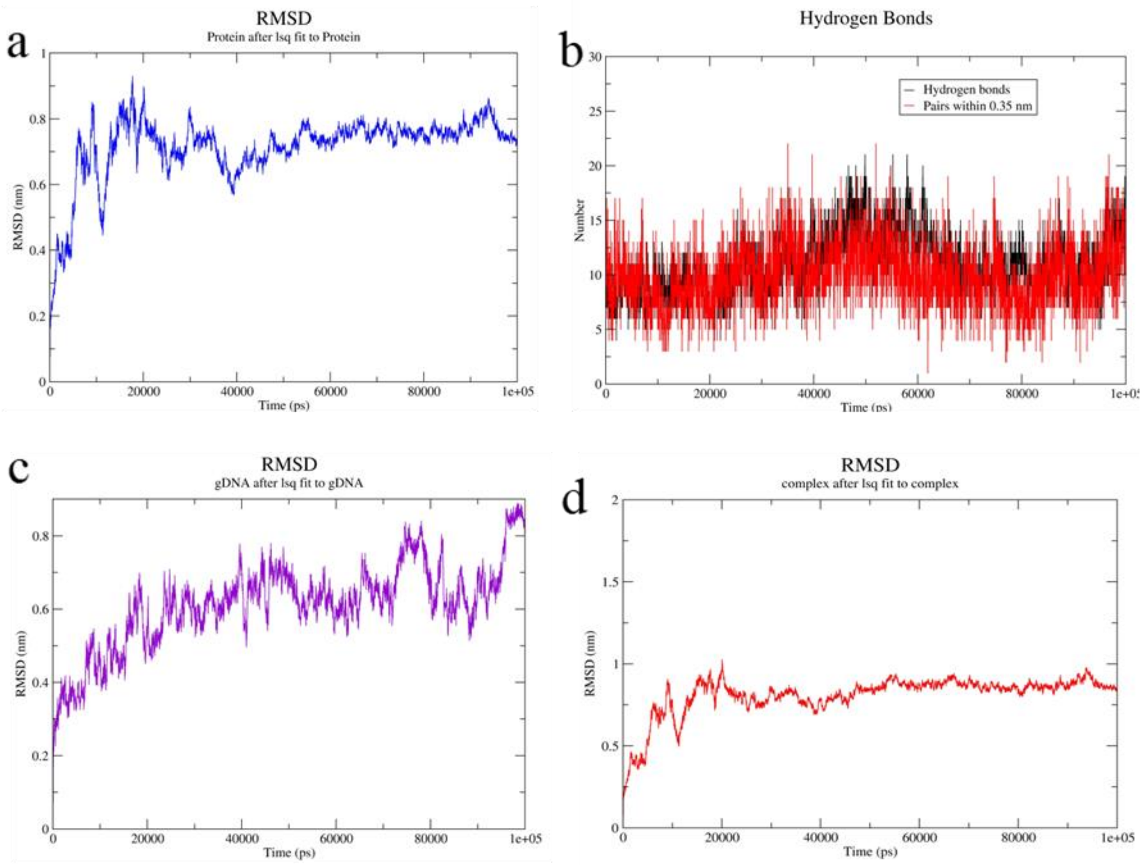

Supplementary Figure 21. **Molecular dynamic simulations of NgAgo protein and guide DNA.** (a) NgAgo protein root mean square deviation (RMSD) curves during molecular dynamic simulations. (b) The number of hydrogen bonds between NgAgo protein and guide DNA during molecular dynamic simulations. (c) The gDNA RMSD curves during molecular dynamic simulations. (d) RMSD curves during molecular dynamic simulations.

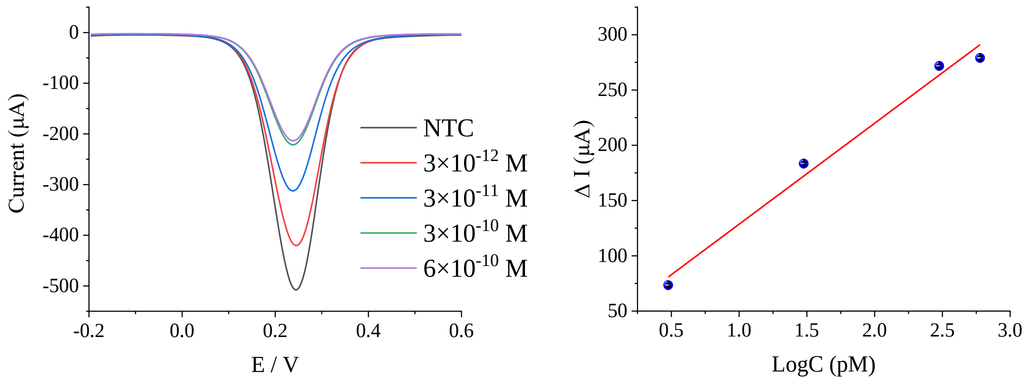

Supplementary Figure 22. **The graphene surface with Ng system for recognizing target EBV DNA, in the range of 0, 3×10^{-12} M, 3×10^{-11} M, 3×10^{-10} M, 6×10^{-10} M, using 0.05 M $[Fe(CN)_6]^{3-/4-}$.**

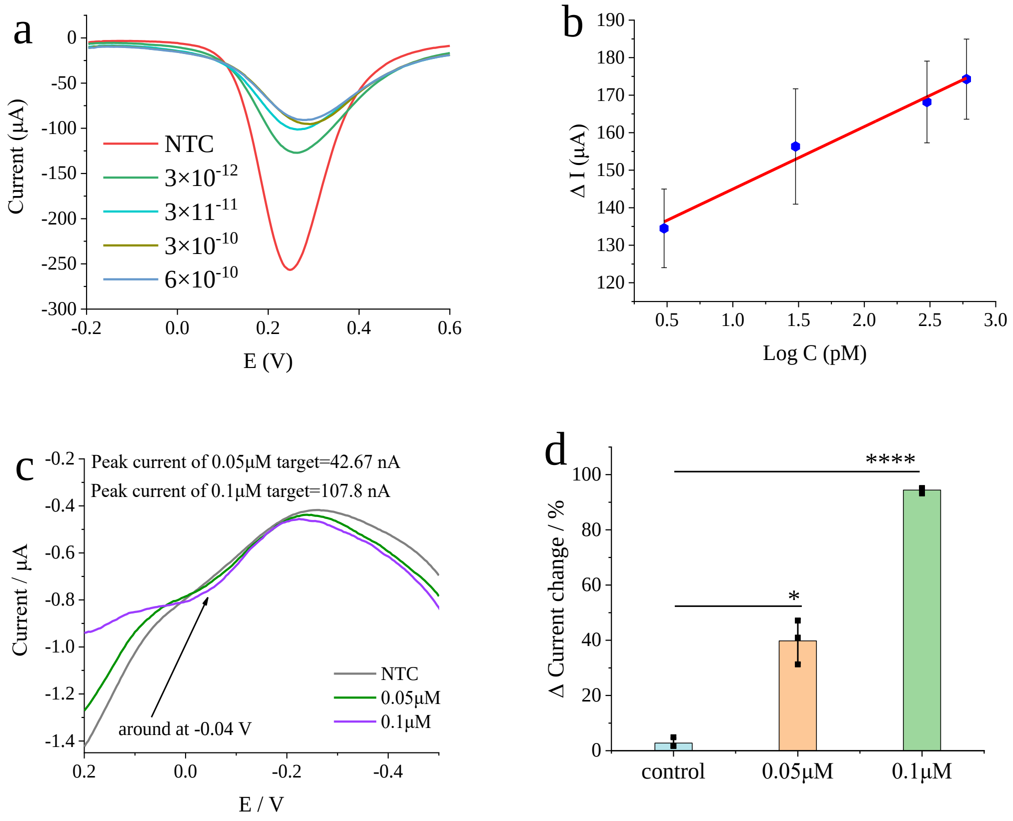

Supplementary Figure 23. **The gDNA3-guided Ng system without graphene surface for recognizing target EBV DNA.** (a) DPV plots of gDNA3-guided Ny system under different concentrations of target DNA, incubation at 37°C for 60 min using 0.05 M $[Fe(CN)_6]^{3-/4-}$. (b) Calibration curve of figure 23(a). (c) DPV plots of gDNA3-guided Ny system for recognizing FAM-labelled target DNA. (d) Current response of figure 23(c), 0.01 M PBS, pH 7.4, incubation at 37°C for 10 min, using two-way ANOVA: *$p<0.05$, **$p<0.01$, ***$p<0.001$, ****$p<0.0001$, p value of 0.01, 0.000088, for 0.05, 0.1 μM groups respectively, data presented as the mean values ± SDs, n=3 independent experiments.

.

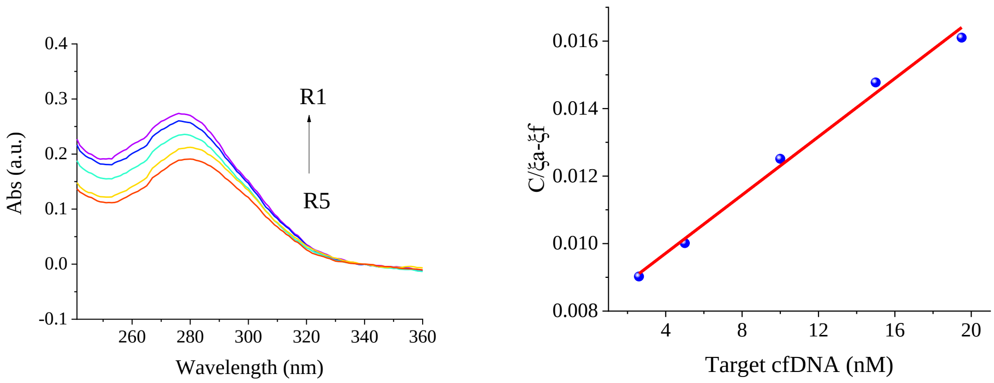

Supplementary Figure 24. **The investigation on the gDNA-guided Ng system binding with target DNA validated by UV-vis spectrum.** R=target DNA concentration : NgAgo/gDNA concentration, R1 to R5 referring to 1.95, 1.5, 1, 0.5, 0.25, respectively. It was found that as the R value increased, the characteristic absorption peaks of NgAgo protein increased, with a slight shift (~ 4 nm), which preliminarily explained that NgAgo-gDNA complex combined with target DNA.

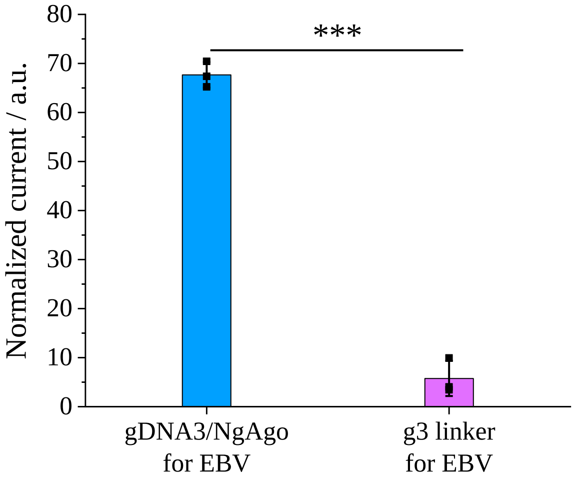

Supplementary Figure 25. **The comparison of gDNA3-guided Ng system and g3 linker for recognizing target EBV DNA, using 0.05 M $[Fe(CN)_6]^{3-/4-}$.** The sequence of g3 linker was a single-stranded DNA oligo, listed in Supplementary Table 1,

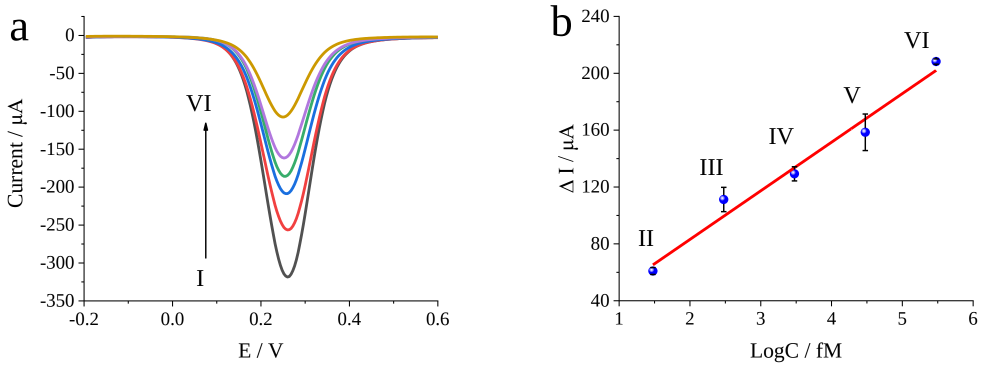

Supplementary Figure 26. **End-point quantitative detection of EBV cfDNA by graphene-TDN-Ng platform.** (a) DPV plots of the platform under different concentrations of EBV cfDNA. (b) Calibration curve. I to VI referring to 0, 3×10^{-14} M, 3×10^{-13}M, 3×10^{-12} M, 3×10^{-11} M, 3×10^{-10}M of target EBV cfDNA, using 50 mM $[Fe(CN)_6]^{3-/4-}$, 37 ° C, 60 min, $\triangle I(\mu A)=34.1938\cdot logC(fM)+14.7224$, (R=0.9873).

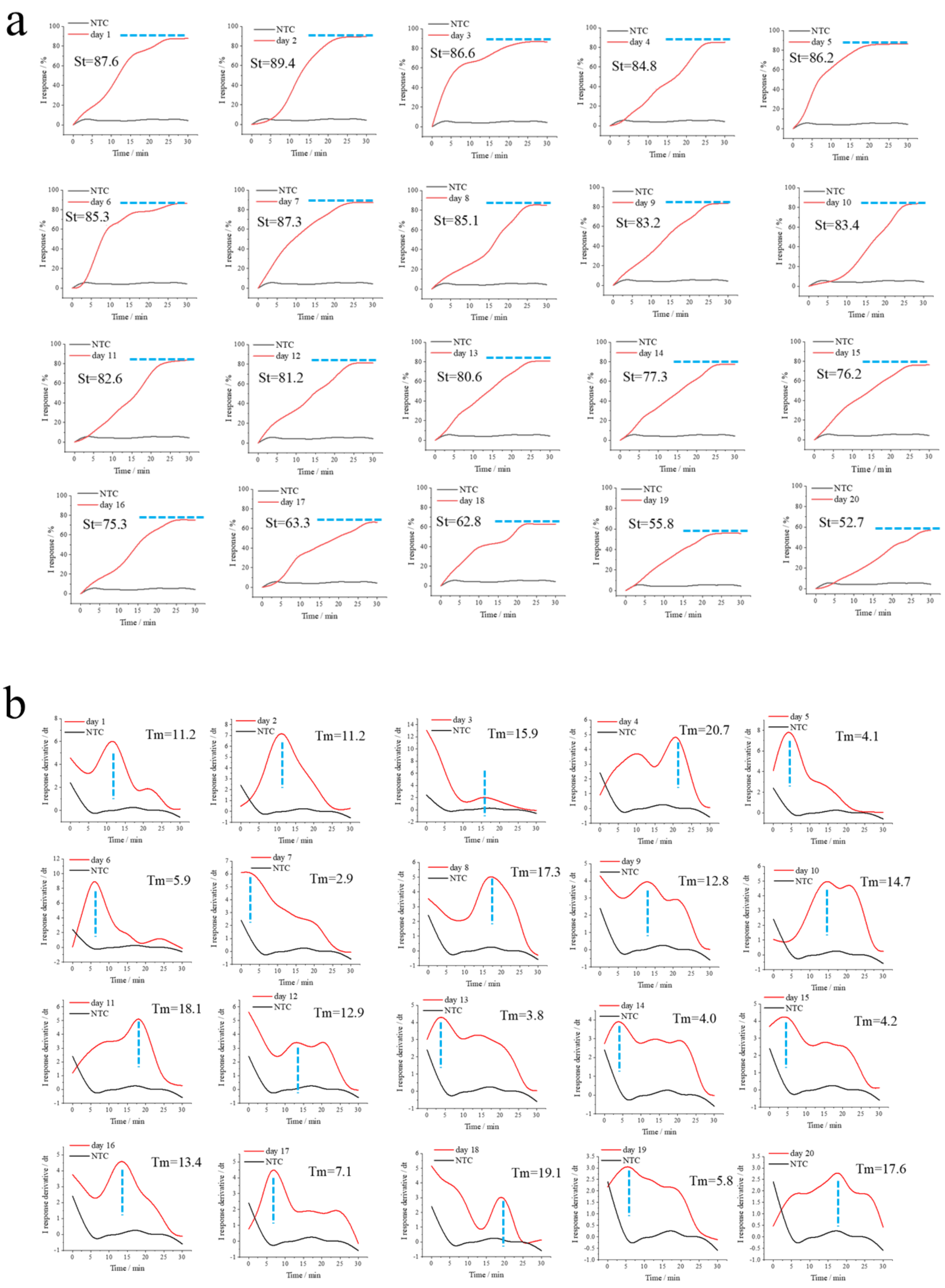

Supplementary Figure 27. **Stable sensitivity tests *in vitro*.** (a) Real-time signal curves for 20 days, n=3 independent experiments. (b) the relative slope of the plots for 20 days, calculated by simple differentiation, n=3 independent experiments. S_t value was defined as signal platform threshold and T_m value was defined as the time threshold corresponding to the maximum of signal response curve derivatives.

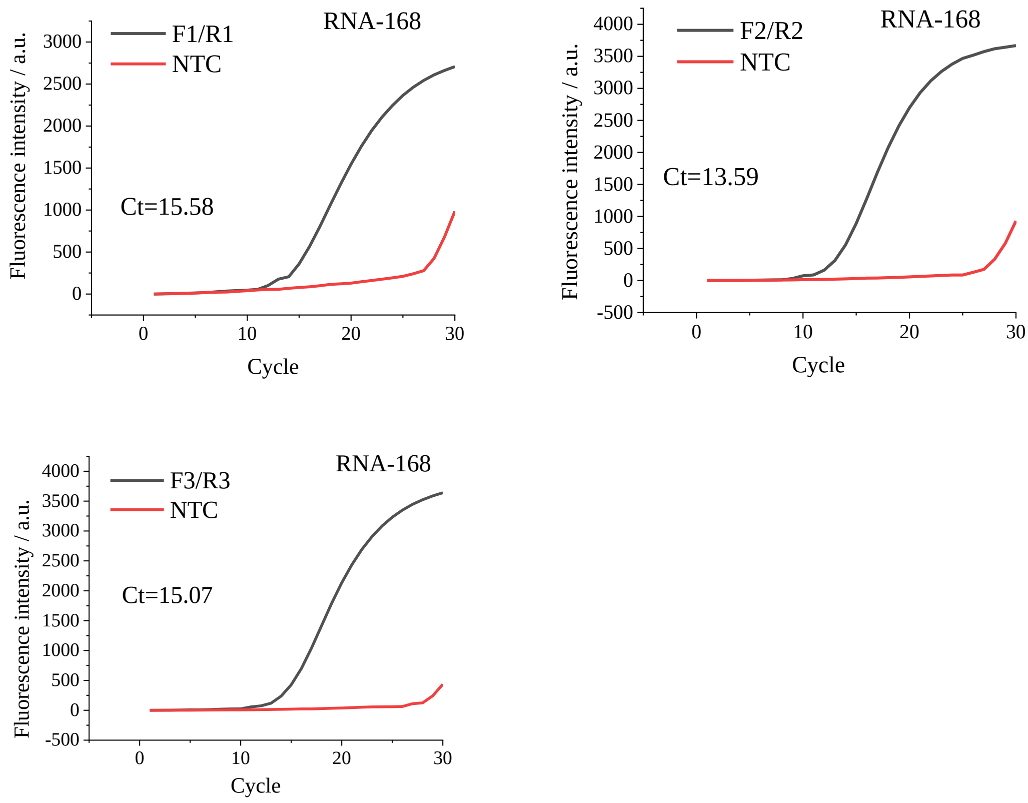

Supplementary Figure 28. **RT-PCR primers screening for RNA-168.**

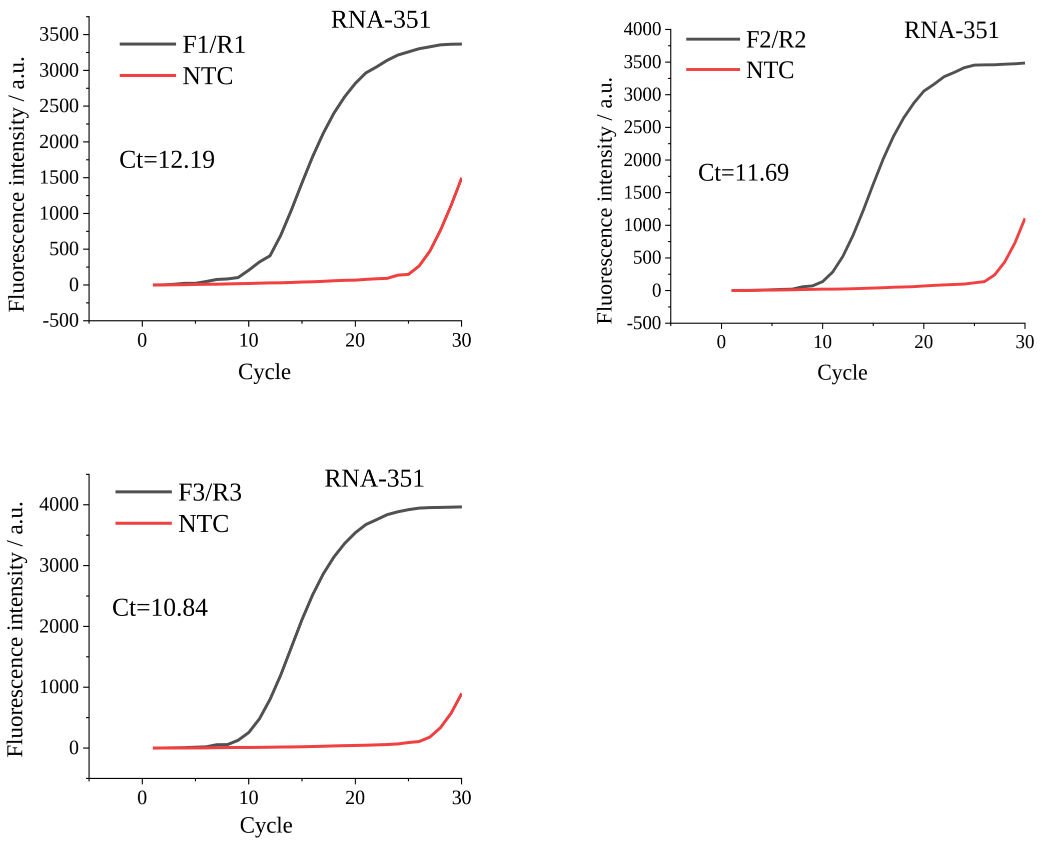

Supplementary Figure 29. **RT-PCR primers screening for RNA-351.**

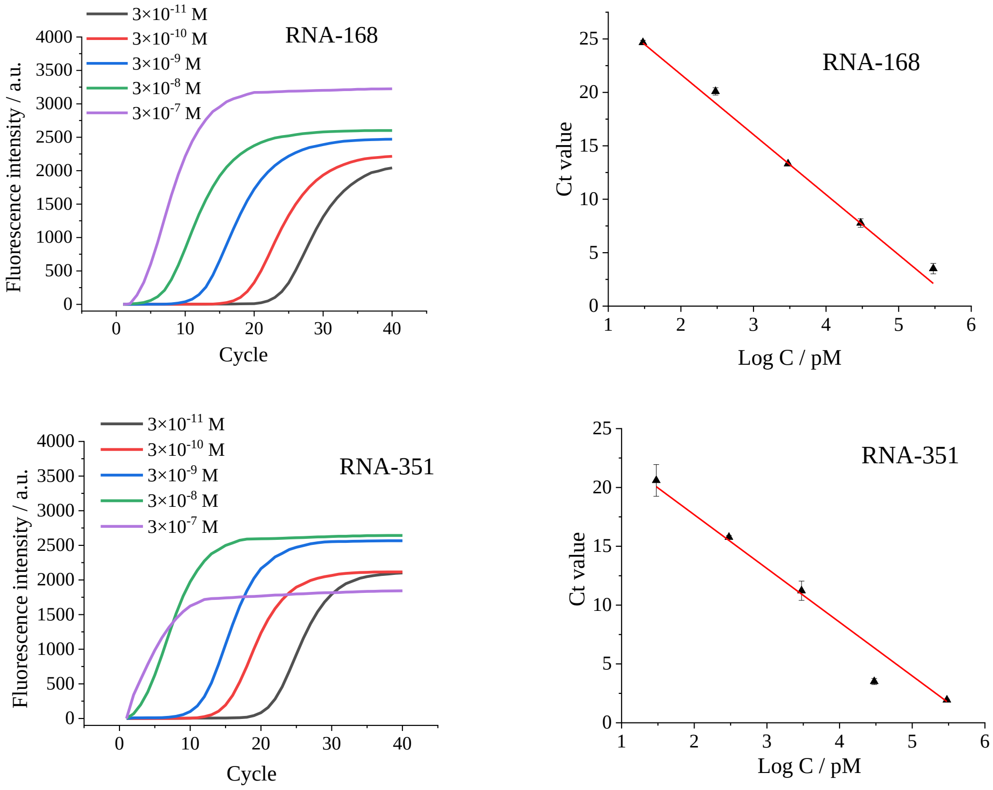

Supplementary Figure 30. **Dynamic curves and calibration of RNA-168 and RNA-351 by RT-PCR.**

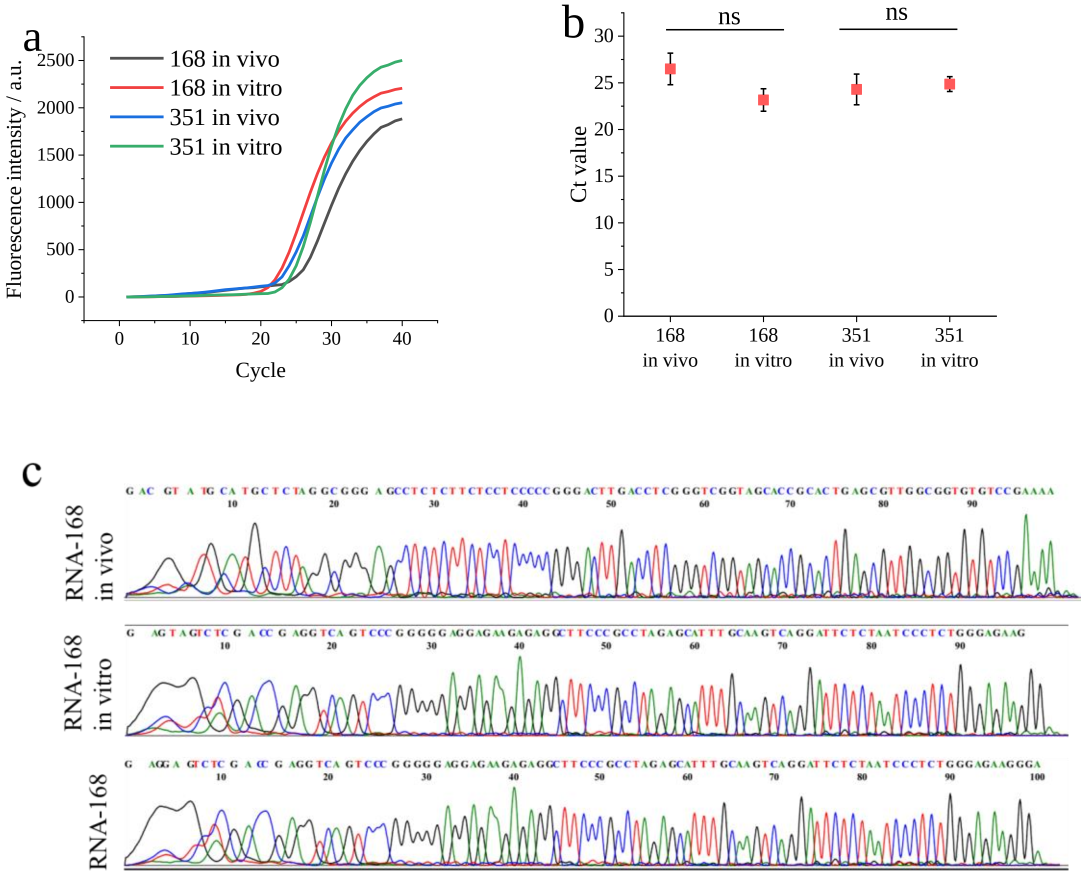
a
Fluorescence intensity / a.u.
2500
2000
1500
1000
500
0
0
10
20
30
40
Cycle
168 in vivo
168 in vitro
351 in vivo
351 in vitro
b
ns
ns
Ct value
30
25
20
15
10
5
0
168 in vivo
168 in vitro
351 in vivo
351 in vitro
c
RNA-168 in vivo
RNA-168 in vitro
RNA-168

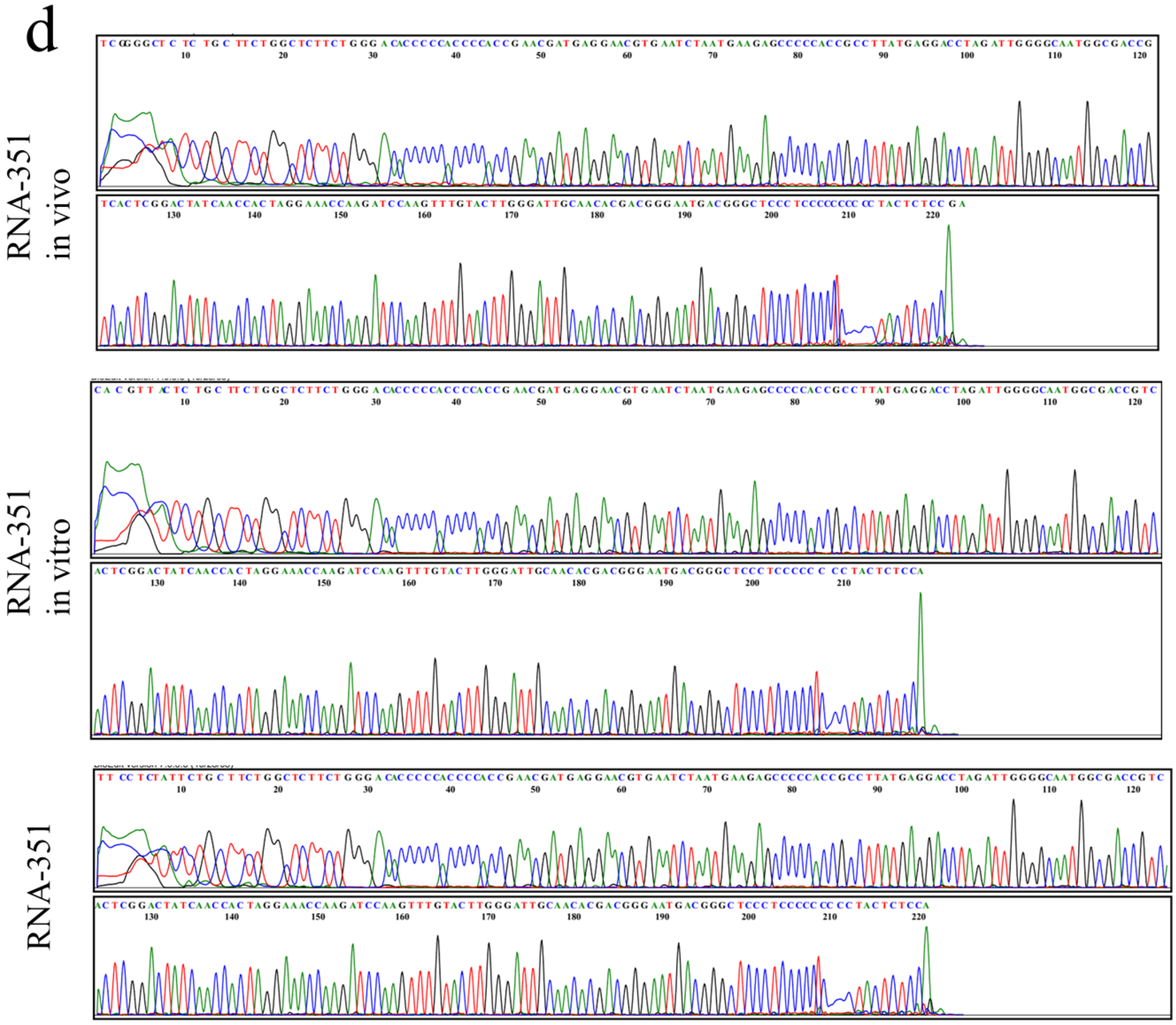

Supplementary Figure 31. **Genotyping of RNA-168 and RNA-351 extracted from CNE-Luc cell lines.** (a) Dynamic curves via RT-PCR. (b) Comparison of Ct value. (c) Next-generation sequencing of RNA-168 groups, including CNE-Luc in vivo, CNE-Luc in vitro, standard samples. (d) Next-generation sequencing of RNA-351 groups, including CNE-Luc in vivo, CNE-Luc in vitro, standard samples.

a

b

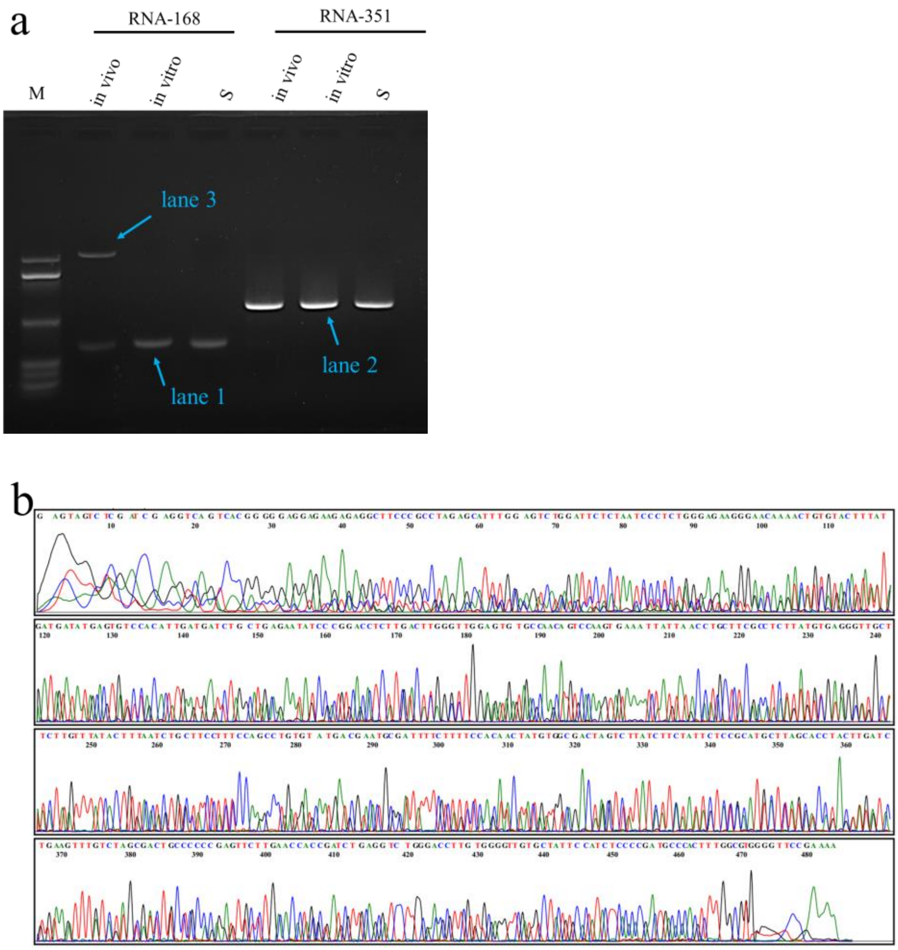

c

	Description	Scientific Name	Max Score	Total Score	Query Cover	E value	Per. ident	Acc. Len	Accession
lane 1	Human gammaherpesvirus 4 isolate AA-43, partial genome	Human gammaherpesvirus 4	154	154	90%	3.00E-33	97.78	171766	ON023030.1
	Human gammaherpesvirus 4 genome assembly, plasmid: 1	Human gammaherpesvirus 4	154	154	90%	3.00E-33	97.78	167492	LR994527.1
	Human gammaherpesvirus 4 genome assembly, plasmid: 1	Human gammaherpesvirus 4	154	154	90%	3.00E-33	97.78	170369	LR994522.1
	Human gammaherpesvirus 4 genome assembly, plasmid: 1	Human gammaherpesvirus 4	154	154	90%	3.00E-33	97.78	167213	LR994505.1
	Human gammaherpesvirus 4 isolate Necker-20, partial genome	Human gammaherpesvirus 4	154	154	90%	3.00E-33	97.78	169388	MT648661.1
	Human gammaherpesvirus 4 isolate Necker-19, partial genome	Human gammaherpesvirus 4	154	154	90%	3.00E-33	97.78	167866	MT648660.1
	Human gammaherpesvirus 4 isolate OLP-5 EBER snRNA gene, complete sequence	Human gammaherpesvirus 4	154	154	90%	3.00E-33	97.78	627	MN921219.1
	Human gammaherpesvirus 4 isolate OSCC-237 EBER snRNA gene, complete sequence	Human gammaherpesvirus 4	154	154	90%	3.00E-33	97.78	640	MN921217.1
	Human gammaherpesvirus 4 isolate OSCC-36 EBER snRNA gene, complete sequence	Human gammaherpesvirus 4	154	154	90%	3.00E-33	97.78	639	MN921216.1
	Human gammaherpesvirus 4 isolate OSCC-57 EBER snRNA gene, complete sequence	Human gammaherpesvirus 4	154	154	90%	3.00E-33	97.78	624	MN921215.1
lane 2	Human gammaherpesvirus 4 strain rMSHJ, complete genome	Human gammaherpesvirus 4	381	381	95%	3.00E-101	99.06	164674	MK973062.1
	Human gammaherpesvirus 4 UPN412_CD56+ DNA, nearly complete genome	Human gammaherpesvirus 4	381	381	95%	3.00E-101	99.06	157546	AP019146.1
	Human gammaherpesvirus 4 isolate ebv27, partial genome	Human gammaherpesvirus 4	381	381	95%	3.00E-101	99.06	169463	MH883769.1
	Human gammaherpesvirus 4 isolate ebv25, partial genome	Human gammaherpesvirus 4	381	381	95%	3.00E-101	99.06	169628	MH883768.1
	Human gammaherpesvirus 4 isolate ebv22, partial genome	Human gammaherpesvirus 4	381	381	95%	3.00E-101	99.06	169502	MH883766.1
	Human gammaherpesvirus 4 isolate ebv17, partial genome	Human gammaherpesvirus 4	381	381	95%	3.00E-101	99.06	168871	MH883763.1
	Human gammaherpesvirus 4 isolate ebv15, partial genome	Human gammaherpesvirus 4	381	381	95%	3.00E-101	99.06	168271	MH883761.1
	Human gammaherpesvirus 4 isolate ebv9, partial genome	Human gammaherpesvirus 4	381	381	95%	3.00E-101	99.06	168523	MH883758.1
	Human herpesvirus 4 isolate 07/331 latent membrane protein 2A (LMP 2A) gene, partial	Human gammaherpesvirus 4	381	381	95%	3.00E-101	99.06	363	JX237782.1
	Epstein-Barr virus LMP2A gene (Fay)	Human gammaherpesvirus 4	381	381	95%	3.00E-101	99.06	447	X81767.1
	Epstein-Barr virus latent membrane protein 2A (LMP 2A) mRNA, complete cds	Human gammaherpesvirus 4	381	381	95%	3.00E-101	99.06	351	M87778.1
lane 3	Human herpesvirus 4 genome assembly sLCL-IM1.17, segment : I	Human gammaherpesvirus 4	134	134	18%	3.00E-26	93.48	172060	LN827583.1
	Human gammaherpesvirus 4 isolate AA-43, partial genome	Human gammaherpesvirus 4	132	132	17%	9.00E-26	94.25	171766	ON023030.1
	Human gammaherpesvirus 4 genome assembly, plasmid: 1	Human gammaherpesvirus 4	132	132	17%	9.00E-26	94.25	167492	LR994527.1
	Human gammaherpesvirus 4 genome assembly, plasmid: 1	Human gammaherpesvirus 4	132	132	17%	9.00E-26	94.25	170369	LR994522.1
	Human gammaherpesvirus 4 genome assembly, plasmid: 1	Human gammaherpesvirus 4	132	132	17%	9.00E-26	94.25	167213	LR994505.1
	Human gammaherpesvirus 4 isolate Necker-20, partial genome	Human gammaherpesvirus 4	132	132	17%	9.00E-26	94.25	169388	MT648661.1
	Human gammaherpesvirus 4 isolate Necker-19, partial genome	Human gammaherpesvirus 4	132	132	17%	9.00E-26	94.25	167866	MT648660.1
	Human gammaherpesvirus 4 isolate Necker-18, partial genome	Human gammaherpesvirus 4	132	132	17%	9.00E-26	94.25	170699	MT648659.1
	Human gammaherpesvirus 4 isolate Necker-16, partial genome	Human gammaherpesvirus 4	132	132	17%	9.00E-26	94.25	169461	MT648657.1
	Human gammaherpesvirus 4 isolate Necker-15, partial genome	Human gammaherpesvirus 4	132	132	17%	9.00E-26	94.25	170445	MT648656.1

Supplementary Figure 32. **Gene-typing of different sequences.** (a) Agarose electrophoresis, 2% agarose, 100 V, 45 min, 1×TAE. (b) Next-generation sequencing for lane 3 sequence. (c) Blast result of lane 1-3 sequences from NCBI database.

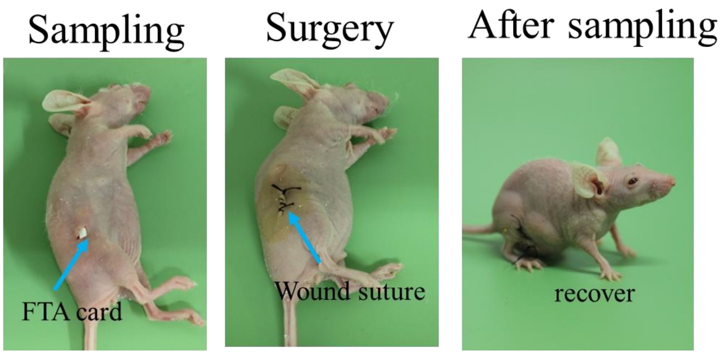

Supplementary Figure 33. **ISF sampling method.** The whatman FTA card was used for collecting ISF samples and extracted by a commercial nucleic acid kit.

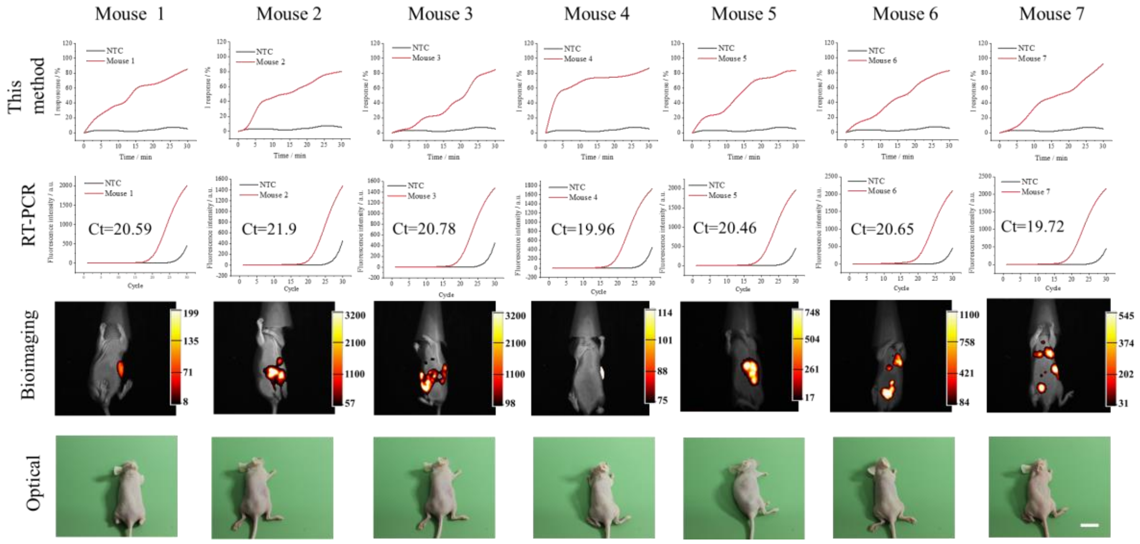

Supplementary Figure 34. **Paralleled demonstrations of mice for RNA biomarkers monitoring.**

Scale bar, 1cm.

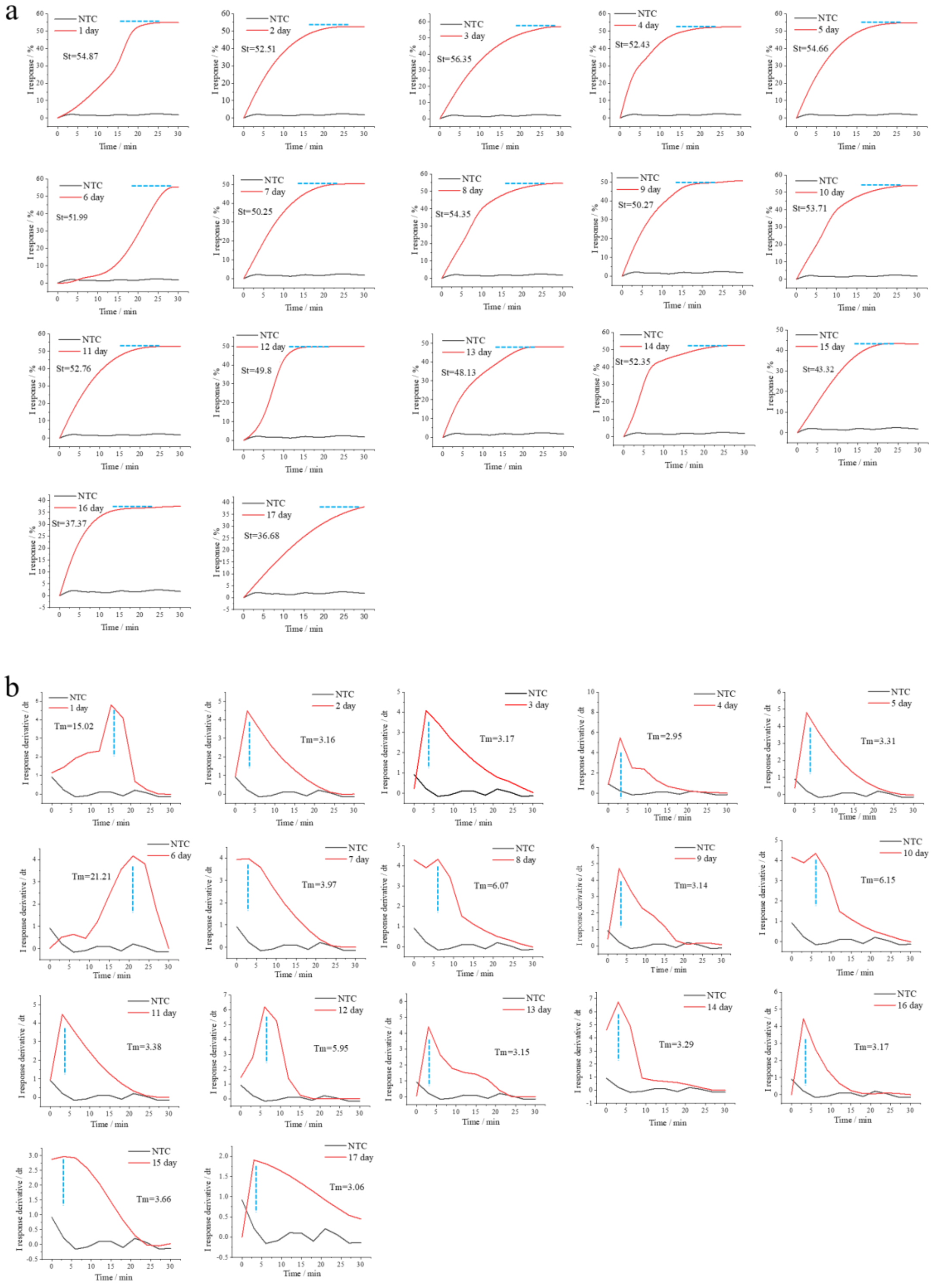

Supplementary Figure 35. **Stable sensitivity tests *in vivo*.** (a) Real-time signal curves for 17 days, n=3 independent experiments. (b) the relative slope of the plots for 17 days, calculated by simple differentiation, n=3 independent experiments. S_t value was defined as signal platform threshold and T_m value was defined as the time threshold corresponding to the maximum of signal response curve derivatives.

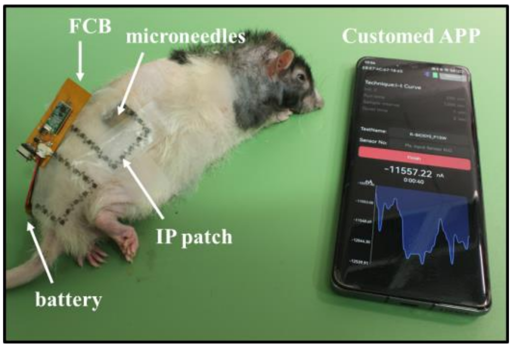

Supplementary Figure 36. **Demonstration of the integrated wearable electronics on immunodeficiency mouse.** The iontophoretic patch (IP patch) was powered by a battery on FCB, and the microneedles detected the target as well as produced signal, and the FCB recorded the data and transmitted it into a mobile device.

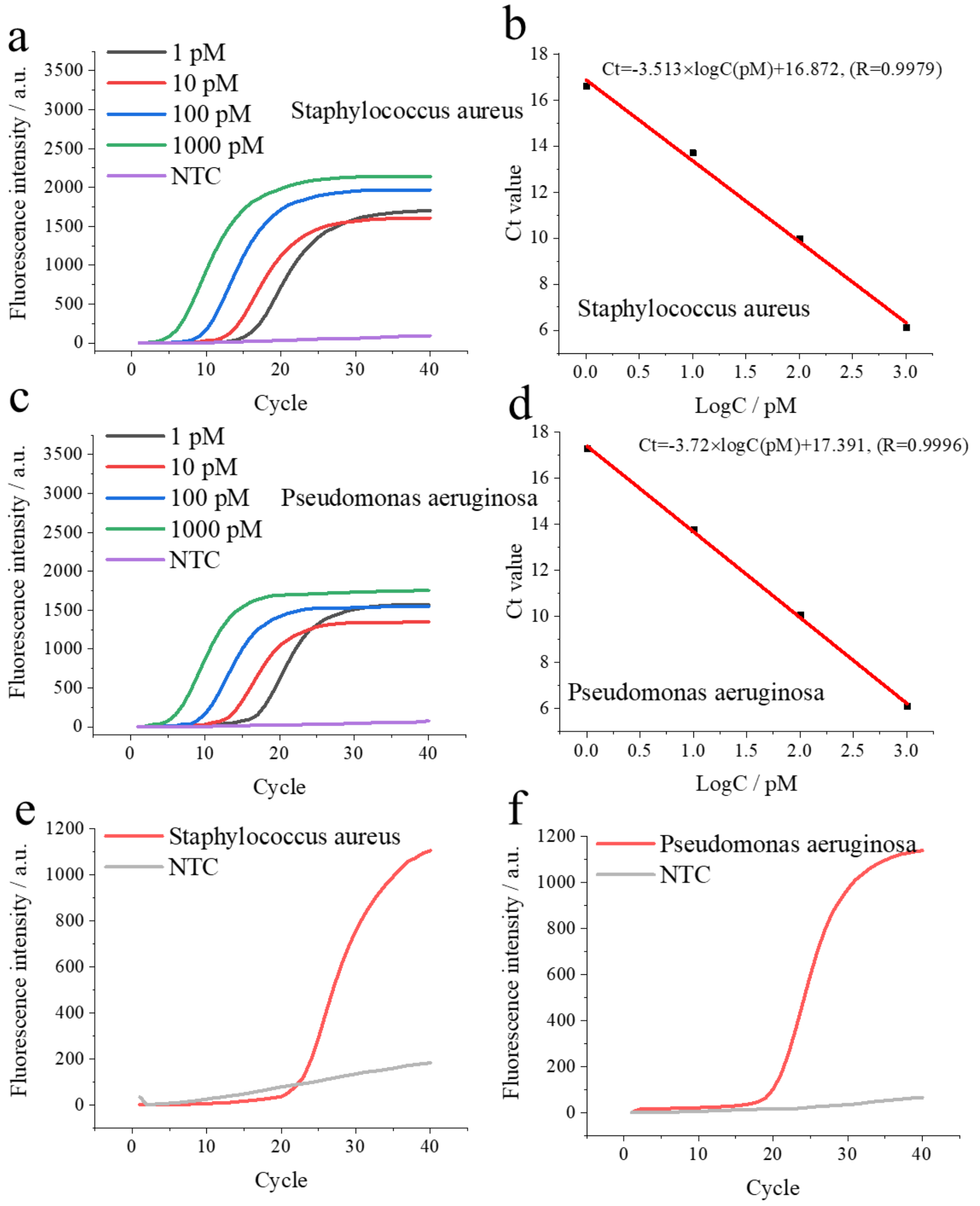

Supplementary Figure 37. **Dynamic curves and calibration of SA and PA by PCR.** (a) and (b) PCR plotsfor nuc gene fragment of SA. (c) and (d) PCR plots for lasR gene fragment of PA. (e) PCR plots for real SA strain. (f) PCR plots for real PA strain.

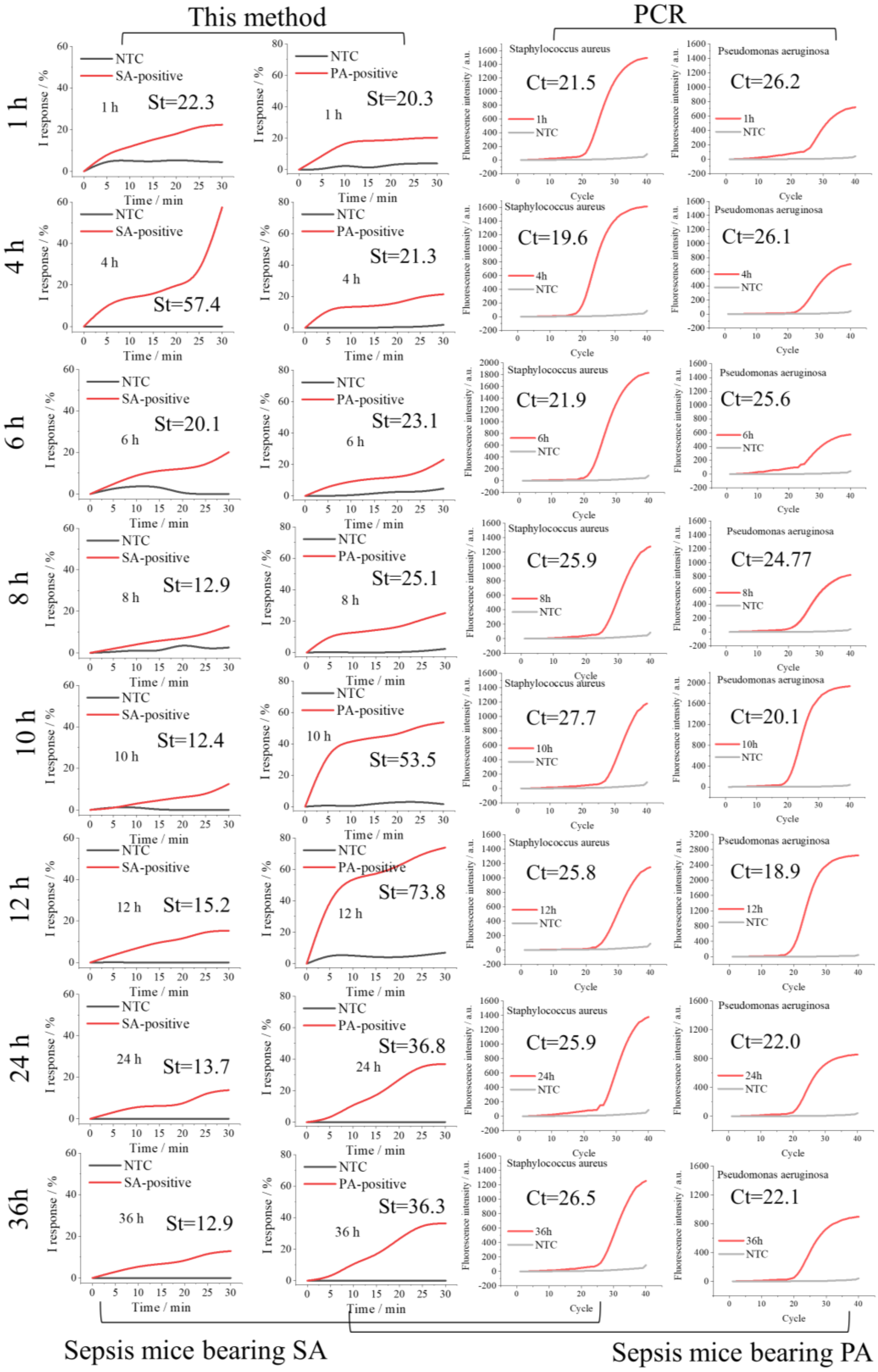

Figure 38. **Raw data of continuous parallel demonstrations on sepsis mice bearing SA and PA strain at different time points for 36 h.**

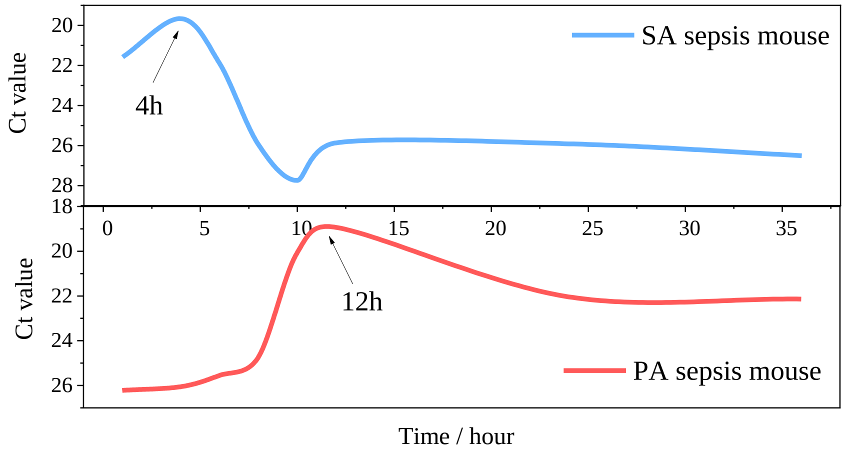

Figure 39. **Paralleled demonstrations on sepsis mice bearing SA and PA strain at different time points within 36 h recorded by gold standard PCR.**

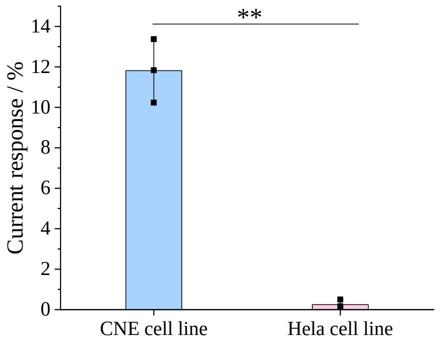

Figure 40. **Selectivity of TDN-Ng platform for different cell lines lysate in vitro.** 1×10^{7} cell/mL cell, incubated at 37 °C for 30 min, analysed by two-way ANOVA: *p<0.05, **p<0.01, ***p<0.001, ****p<0.0001, data presented as the mean values ± SDs, n=3 independent experiments, repeated time=3, using 50 mM $[Fe(CN)_6]^{3-/4-}$, CNE as target group, Hela as non-target group.

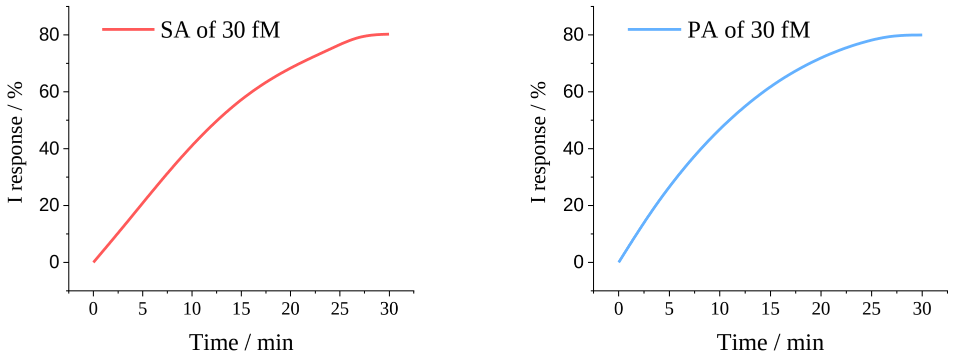

Supplementary Figure 41. **The application of TDN-Ng sensor for real-time monitoring SA and PA target DNA in vitro, PBS (0.01 M, pH 7.4), 37°C, reverse iontophoresis of 10 V.**

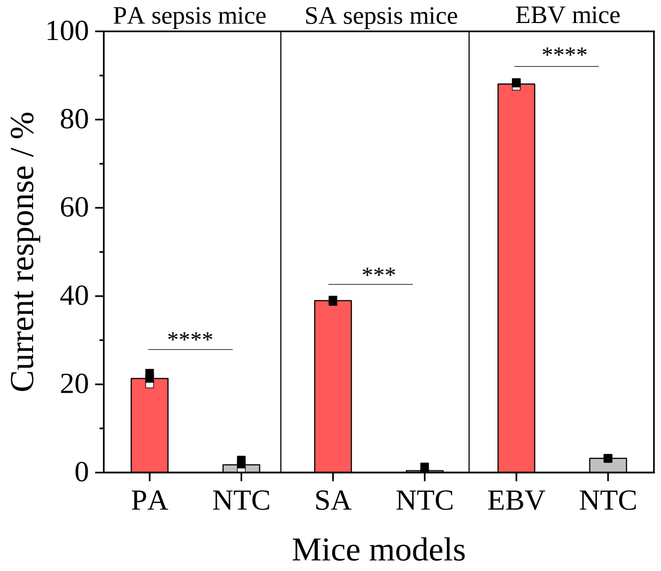

Supplementary Figure 42. **Specificity detection in vivo of the TDN-Ng MN patch for three different mice models, at the 4h time point.** NTC group referring to other two non-target models.

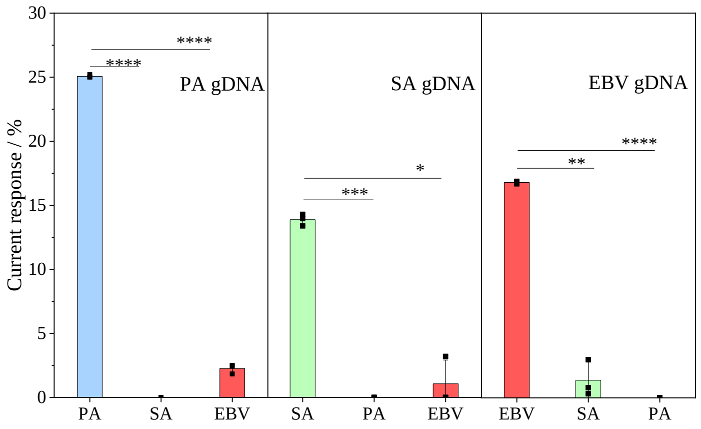

Supplementary Figure 43. **Selectivity of the sensor with scrambled guide DNA under different nucleic acid of 0.3 nM in vitro.** The sensor was incubated in samples for 30 min, analysed by two-way ANOVA: *p<0.05, **p<0.01, ***p<0.001, ****p<0.0001, data presented as the mean values ± SDs, n=3 independent experiments, repeated time=3. using 50 mM $[Fe(CN)_6]^{3-/4-}$. NTC group referring to other two non-target models.

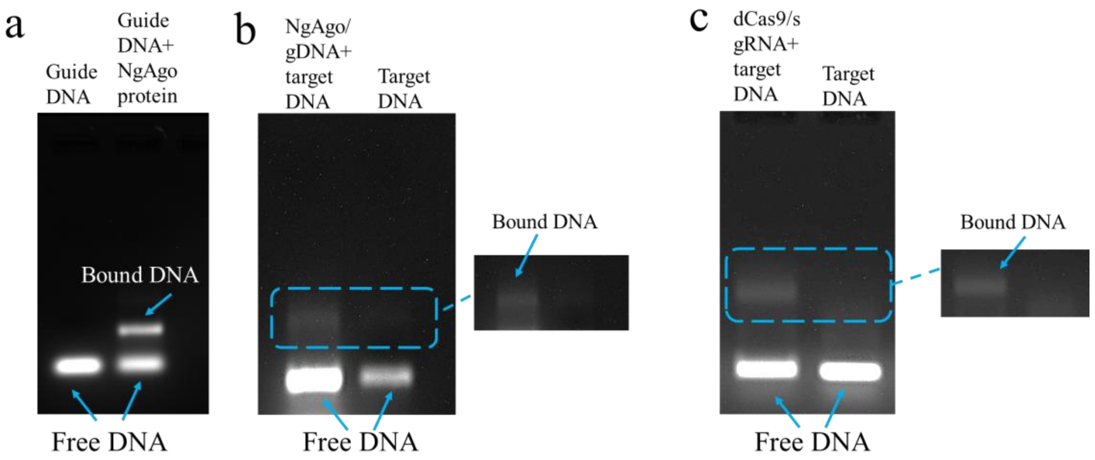

Supplementary Figure 44. **Electrophoretic mobility shift assay (EMSA) of Ng system.** (a) EMSA of guide DNA with NgAgo protein, 2% agarose of no dye, 100V, 35min, 1×TAE buffer, exposure time of 6s100ms. (b) EMSA of Ng system, 2% agarose of no dye, 100V, 45min, 1×TAE buffer, exposure time of 6s100ms. (c) EMSA of CRISPR-dCas9, 2% agarose of no dye, 100V, 45min, 1× TAE buffer, exposure time of 6s100ms.

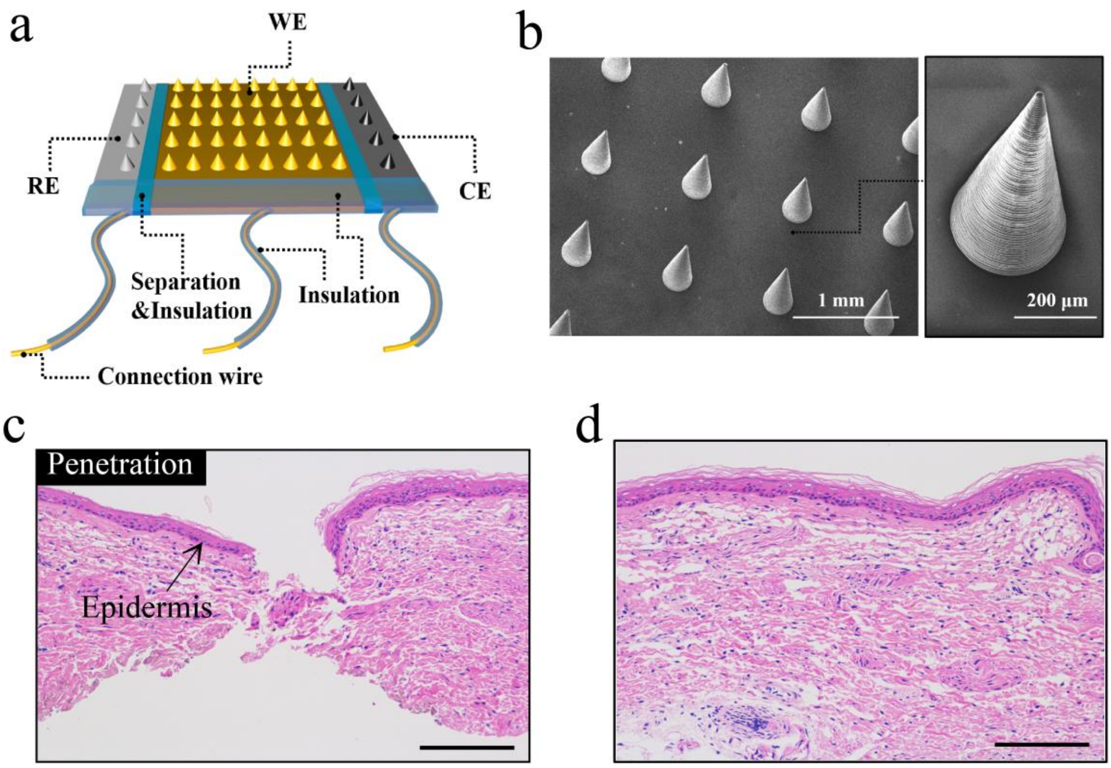

Supplementary Figure 45. **Characterization of the MN patch.** (a) Schematic of the integrated MN patch. RE, WE, CE refer to the reference, working and counter electrodes, respectively. (b) SEM photograph of the microneedles. Histology from piglet skin penetrated by the MN (c) and NTC group (d), stained with HE, the black arrow refers to the inserted direction, scale bar, 100 μm.

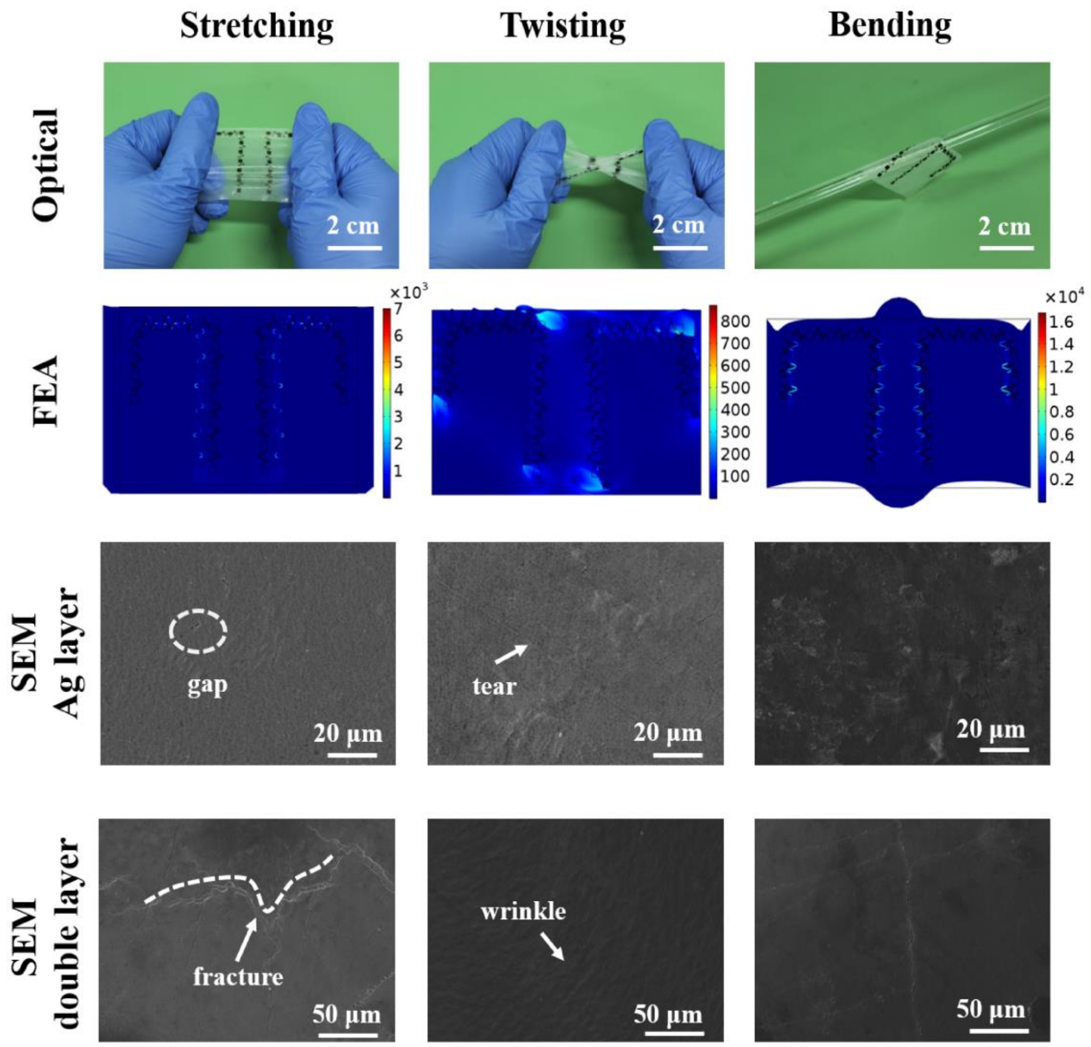

Supplementary Figure 46. **Characterization of the TPU patch.** Optical images, finite elemental analysis by COMSOL Multiphysics, SEM of the dual-layered printed wearable patch under variable mechanical distortions, involving stretching, twisting, and bending.

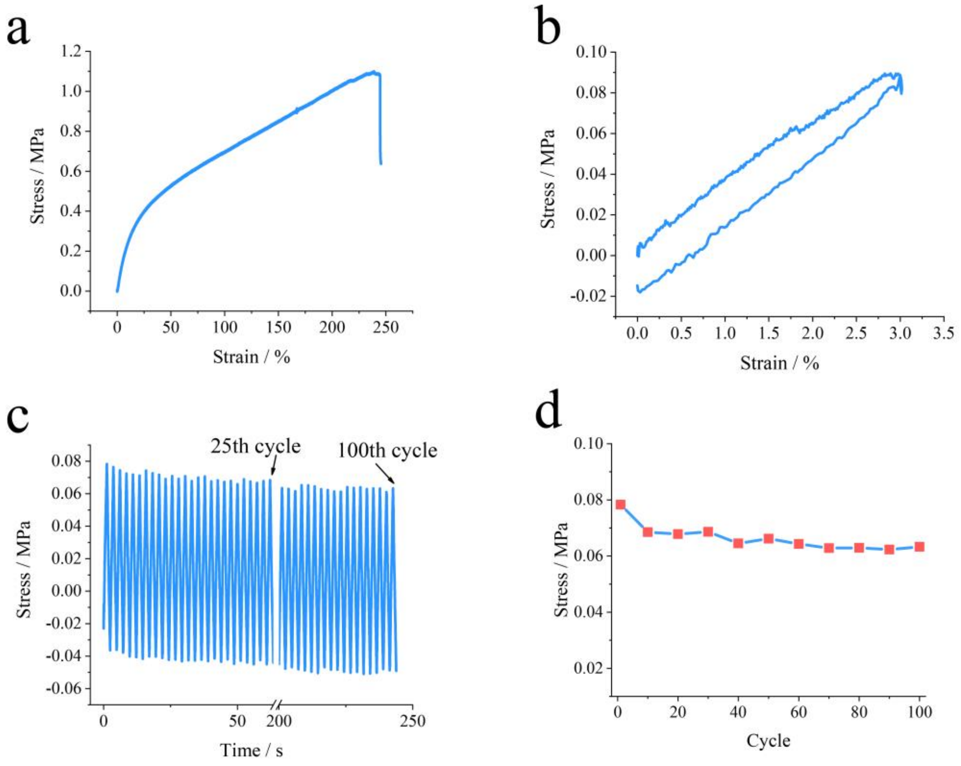

Supplementary Figure 47. **Mechanical performance of the TPU patch.** (a) Strain versus stress plot for the functional TPU film. (b) One stretch-release cycle with 10% strain for the functional TPU film. (c) Stress variation of the functional TPU film in a 100-cycle test with a strain of 4%. (d) Stress changes of the functional TPU film every ten cycles.

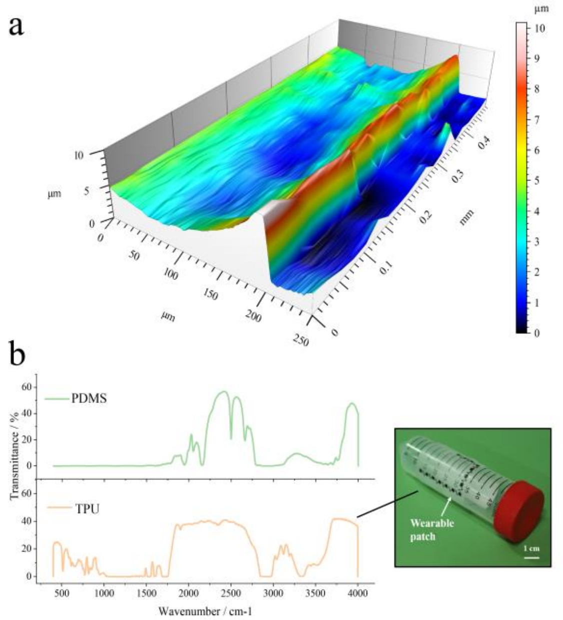

Supplementary Figure 48. **Surface characterization of the printed TPU patch.** (a) The 3D view of the dual-layered printed TPU film analysed by stylus profiler, 0.05 mg loading force, 0.1 mm/s scanning speed. (b) The transparency of two different functional films (PDMS and TPU) was analysed by a Fourier transform infrared spectrometer.

Supplementary Note 1. Comparison on different Ng/gDNA modified graphene bio-interfaces based on double-stranded DNA ladder.

The experimental results are shown in Supplementary Figure 12. Through repeated duplication tests (n=3, repeated time=3), the results showed that as DNA ladder length increased, the current response of bulk potential continued to increase, while signal response of ladder 20 group was the largest. When the ladder length continued to increase, the current response gradually decreased. We found that the current response was basically weak, exceeding the 40-bp ladder modification, and this phenomenon tended to be steady.

Supplementary Note 2. Real-time monitoring ability of TDN-Ng bio-interfaced microelectrode.

To explore the sensitivity and real-time monitoring for target DNA, a simple biosensor was constructed based on graphene-TDN-Ng via a commercial microelectrode. The microelectrode was used to continuously monitor target DNA in a skin chip containing stimulated ISF (37°C, 0.01 M PBS, pH 7.4), under reverse iontophoresis of 10V. In the range of 3×10^{-16} M to 3×10^{-13} M of target EBV cfDNA, there was a certain linear relationship between the relative I response and concentration of Ng complex, with a linear equation of I response (%) = 17.8143·logC(fM)+60.2025, (R=0.9993), with a detection limit of 3×10^{-16} M. And positive groups had obvious T_m value, while there is none in NTC group. Compared with previous work, the detection limit had lowered by two orders of magnitude on wearable platform.

Supplementary Note 3. Theory and deduction of Gaussian Box based on Gouy-Chapman-Stern model.

Firstly, we need to discuss on the improved biosensing sensitivity resulting from TDN modified bio-interface. In this system, the biosensing layer was constructed on the surface of graphene which is a typical 2D material with Dirac cones, which particularly leads to ultrahigh carrier mobility. Then, TDN and Ng system was successively anchored on the surface of graphene. It's envisaged that there are two phases to form electrical double layers: (1) phase A including TDN, Ng system, recognized target nucleic acid on graphene biointerfaces channel; (2) phase B including ISF bulk microenvironment. It was a well-organized layer that could offset the difference in charge between the phase A and phase B. Then, Donnan potential occurred when the ion permeable layer separated two kinds of ions, as depicted in following Nernst formula equation 1,

$$\phi_D = \frac{RT}{F}\text{In}r_D \quad (1),$$

where r_D referred to Donnan equilibrium constant.

Additionally, the situation of the system is more complex. Various ions have a certain size, and it's impossible to get closer to the surface than its radius. The ions remain solvent, then the thickness of the initial solution shell needs to be added to the radius of the ions, and additional increments need to be considered for the phase A on the conducting microneedle electrode. In other words, the ion center at the distance of X_2 can be deemed as the closest layer. Here, a concept needs to be introduced that X_2 is called the outer Helmoholtz plane (OHP). Because there is the same field strength between the surface of the microneedle and OHP, the potential distribution of the tight layer is linear, so the total potential equation 2 of the electrical double layers is shown as follows,

$$\phi_0 = \phi_2 - \left(\frac{\mathrm{d}\phi}{dx}\right)_{x=x_2} x_2 \quad (2).$$

Then, hereby we need to introduce a concept of Gaussian Box in Supplementary Figure 6, which is based on the Gouy-Chapman-Stern model. One end of the box is located on the graphene interface and the other end is perpendicular. The box contains all the charge in the tight layer and the diffuse layer. According to the Gaussian theory, the total charge can be described in equation 3 below,

$$q = \varepsilon\varepsilon_0\left(\frac{\mathrm{d}\phi}{dx}\right)_{x=0}\int_{surfacce}^{end} dS \quad (3).$$

From the theory of Gaussian Box, it's known that the total potential of electrical double layers is the maximum at the end of this box, as following,

$\left(\frac{\mathrm{d}\phi}{dx}\right) = 0$, then $\phi_0 = \phi_2$.

Then, it needs to discuss why TDN-17 is the optimal nano-scaffold in this system. To sum up, the potential in electrical double layers largely depends on Debye length, which measured the charged carried in solution as well as the continuous spatial range of electrostatic effect. Within Debye length, charged substance in electrical double layers output current response. If the electrical double layers excel Debye length, the biosensing signal output might be influenced. Debye length equation 4 is followed below,

$$\lambda_D = \sqrt{\frac{\varepsilon_0\varepsilon_r K_B T}{2N_a e^2 I}} \quad (4),$$

where, λ_D is the Debye length; ε_0 is the permittivity of free space; ε_r is the electrical double layers constant; K_B is Boltzmann's constant; T is Kelvin temperature; N_a is Avogadro's number; e is elementary charge; I is the electrolyte ionic strength.

Based on Gaussian Box theory and Debye length, it can be speculated that ladder 20 and TDN-17 might be the optimal among these comparison groups. Meanwhile, in some previous reports[1], when the lateral spacing of interfaced TDN reached 6 nm, the effective quantity of TDN probe was the uttermost, up to $\sim 11\times10^{10}$. The electrical signal of TDN-interface was depended on both of spatial height and effective probe quantity. Thus, we chose TDN-17 as the optimal nano-scaffold in the subsequent experiments, instead of TDN-26.

Supplementary Note 4. RMSD for NgAgo protein and guide DNA during molecular dynamic simulations.

In Supplementary Figure 21, it showed that the representative simulated model of NgAgo-gDNA complex rapidly remained stable after 30 ns, until the whole simulation process. It can be indicated that the formed complex consisting of engineered NgAgo protein and gDNA was a well-defined stable structure. We further investigate the RMSD curves of gDNA and NgAgo protein individually, showing that the complex was highly stable through formed stable hydrogen bonds between gDNA and NgAgo protein during the whole simulation.

Supplementary Note 5. Investigation on the binding ability of gDNA3-guided Ng system.

To eliminate the interference of graphene, we constructed a simple electrochemical biosensor to verify the binding ability of gDNA3-guided Ng system in different concentrations of target DNA. Firstly, in Supplementary Figure 23a and 23b, it showed that the modified membrane consisting of gDNA3-guided Ng system could recognize target EBV cfDNA, producing a decrease in peak current in $[Fe(CN)_6]^{3-/4-}$ buffer. Meanwhile, target sequences (S1 and S2 for Ng in Supplementary Table 1) were previously labelled with FAM probe, which had a characterized oxidation peak at ~-0.04V. This bulk characterized peak of FAM probe was utilized to further investigate its binding ability. In Supplementary Figure 23c and 23d, the peak current increased with the increasement of target sequences bound with gDNA3-guided Ng system, because the more S2-FAM sequence, the higher current response. These results supported this concept.

Supplementary Note 6. Theory on the TDN-Ng system reaction rate.

We found that the reaction rate of real-time monitoring was faster on the interface loaded with TDN through experiments. We conducted preliminary mechanism studies hereby. The general behavior followed by the adsorption of species i from the solution onto the electrode surface is similar to the electrode reaction behavior. If the surface adsorption rate is fast, an equilibrium can be established on the electrode surface, and the amount of adsorbed species $\Gamma_i(t)$ in a given time is related to the electrode surface adsorbate concentration $C_i(0,t)$ through an appropriate isotherm. The number of adsorbed species during a certain time (t) is related to the flux (i) on the electrode surface as follows,

$$\Gamma_i(t)=\int_0^t D_i\left[\frac{\partial C_i(x,t)}{\partial x}\right]_{x=0} dt,$$

In the TDN-Ng system, nucleic acid was preliminarily enriched by reverse iontophoresis, so it could be regarded as an increasing adsorption rate by stirring the solution. For the above-mentioned linear isothermal formula, it is as follows.

$$\frac{\Gamma_i(t)}{\Gamma_i}=1-\exp\left(\frac{-m_i t}{b_i}\right)$$, where m_i is the mass transfer coefficient.

Therefore, for the TDN-Ng system, compared with the none-TDN interface, the number of recognition receptor NgAgo is relatively reduced at the interface, since TDN regulated the spatial ordered interfaces. For the same concentration of target sample, given that the adsorption amount remains unchanged, b_i becomes smaller, and t also becomes smaller. Thus, the reaction time is shortened.

Supplementary Note 7. Discussion on TDN-Ng interface for recognizing target DNA by end-point method.

According to Supplementary Figure 15, we constructed the interface of TDN/Ng according to the scheme, and performed interface scanning for target DNA of different concentrations in the simulated ISF (PBS, 37 ° C, pH 7.4, 0.01M). As a result, we found that as the target concentration increased, the current value of the oxidation characteristic peak increased, and at the same time, a slight displacement of characterized potential occurred. It was moved to 0.284V. It can also be seen from the data that the current density also increases with the target concentrations. Preliminary, it can infer that after the Ng system recognition of the target DNA is recognized by the interface, the capacitor changed and the current increased due to the changes in the electrical double-layered structure on the interface. Thus, we plan to use this feature oxidation peak to test the real-time curve.

On the basis of the characterized peak exploration, in order to explore whether this oxidation peak has the ability to quantitatively detect target DNA, we used the oxidation peak current of the to explore the current response to different concentration targets. Under different target DNA concentration of 0, 3×10^{-14} M, 3×10^{-13} M, 3×10^{-12} M, 3×10^{-11} M, the prepared TDN-Ng interface was used for target identification in simulated ISF (PBS, 37 ° C, pH 7.4, 0.01M) for 60 min. There is a certain linear relationship between the current signal and target DNA concentration, and the linear equation $\triangle I(\mu A)=0.0149 \cdot \log C(fM)+0.0033$, (R=0.9655). The characteristic peak current increases with the increasement of target concentration, which is of significance for the following real-time methods.

Supplementary Note 8. Primer screening, calibration, gene-typing for EBV RNA

All the primers for RNA-168 and RNA-351 were designed by Primer Premire software, and used for basic RT-PCR experiments for the determination of 3 nM target RNA respectively. And target RNA sequences were selected from NCBI website, including RNA-168 (GenBank: GU205107.1) and RNA-351 (GenBank: M87778.1). In Supplementary Figure 30, one key criterion for screening primers is that forward and reverse primers should have the ability to distinguish positive and NTC group, besides considering Ct value. Thus, we chose F3/R3 of RNA-168 and F3/R3 of RNA-351 as the optimal for the subsequent experiments.

In Supplementary Figure 31, a linear relationship was observed between change in ΔC_t and two target RNA concentration (C) in the range of 3×10^{-11} to 3×10^{-7} M with two equations of $\Delta C_t = -5.6249\cdot\log C(pM)+32.9301$ (R=0.9974) for RNA-168, $\Delta C_t=-4.5665\cdot\log C(pM)+26.8139$ (R=0.9917) for RNA-351, respectively.

To verify whether target RNA was existent in CNE cell lines, the extracted CNE in vivo and in vitro samples were applied for RT-PCR with its optimal primers set. In Supplementary Figure 32a-b, it could be seen that all these four groups had “S” shaped plots during RT-PCR process, whilst there was no significance difference among four groups in terms of Ct value. Additionally, next-generation sequencing results in Supplementary Figure 32c-d were further implied the indeed existence of target RNA in CNE cell lines.

Interestingly, according to agarose electrophoresis in Supplementary Figure 33a, we found that an obvious band at ~500 bp in 168 in vivo (denoted as lane 3), while none in 168 in vitro. Thus, lane 3 was analyzed by next-generation sequencing and blast. We found that the lane 1 (~168 bp of length) was a part of lane 3 (~500 bp of length), demonstrating that the lane 1 of RNA-168 sequence was an RNA fragment and released in vitro from CNE cells, while lane 3 of 500-bp RNA were unable to release in vitro due to its large molecular mass. All the blast results were listed in Supplementary 33c.

Supplementary Method 1. Fabrication of the biosensor based on dsDNA ladder-Ng system

The functionalization steps are as followed: (i) 100 μL 0.2 mg/mL carboxyl graphene (nanoflakes, XFNANO Co., Nanjing) dispersed in 0.1% chitosan was immediately drop-casted on gold electrode surface (diameter of 2 mm) and placed in the oven for 30 min under 60 °C. (ii) Carboxyl group of graphene was activated by 200 μL mixed solution of EDC (200 mM): NHS (50 mM) with a volume of 100 μL:100 μL in 100 μL 100 mM 2-morpholinoethanesulfonic acid buffer (all provided by Aladdin Co., Shanghai) for 60 min under 37 °C. (iii) To prepare different length of dsDNA ladder, 10 μL oligo A (100 μM) and 10 μL oligo B (100 μM) was added into the reaction buffer containing 60 μL ddH_2O and 20 μL hybridization buffer (purchased from Beyotime corporation, Shanghai), and the mixture was incubated in a water bath under 95 °C for 2 min. Then, turned off the water bath, and the it was cooled down to room temperature. (iv) To stabilized dsDNA ladder on the surface, 40 μL of the prepared dsDNA ladder was incubated on the surface of the gold electrode under 37°C for 60 min. (v) To anchor Ng protein, 15 μL of Ng protein (0.7 μM) was added into the above-mentioned 135 μL EDC/NHS solution, and incubated under 37 °C for 120 min. (vi) 1% BSA blocked the non-specific active sites for 60 min under 37 °C. (vii) The electrode was incubated in 1 μM guide DNA (provided by Sangon corporation, Shanghai) for 60 min under 37 °C. (viii) The electrode was washed by 2 mM $MgCl_2$ for 1 min.

Supplementary Method 2. Fabrication of the biosensor based on Ng system for recognizing cfDNA and RNA

The functionalization steps are as followed: (i) the gold electrode (diameter of 2 mm) was incubated in 0.12 M thioglycolic acid (TGA, purchased from Aladdin corporation, Shanghai) under 37 °C overnight (>12 h). (ii) Carboxyl group was activated by 200 μL mixed solution of EDC (200 mM): NHS (50 mM) with a volume of 100 μL:100 μL in 100 μL 100 mM 2-morpholinoethanesulfonic acid buffer (all provided by Aladdin Co., Shanghai) for 60 min under 37 °C.. (iii) To anchor Ng protein, 15 μL of Ng protein (0.7 μM) was added into the above-mentioned 135 μL EDC/NHS solution, and incubated under 37 °C for 120 min. (iv) 1% BSA blocked the non-specific active sites for 60 min under 37 °C. (v) The electrode was incubated in 1 μM guide DNA (provided by Sangon corporation, Shanghai) for 60 min under 37 °C. (vi) The electrode was washed by 2 mM $MgCl_2$ for 1 min. (vii) After incubated in different concentrations of samples, the electrode was washed by ddH_2O for 3 min.

Supplementary Table 1 Nucleic acid sequences in the experiments.

	Sequence (5' - 3')
F10	NH_2-TTTTTTTTTT
R10	COOH-AAAAAAAAAA
F20	NH_2- TTTTTTTTTTTTTTTTTTTT
R20	COOH-AAAAAAAAAA AAAAAAAAAA
F30	NH_2-TTTTTTTTTTTTTTTTTTTTTTTTTTTTTT
R30	COOH-AAAAAAAAAAAAAAAAAAAAAAAAAAAAAA
F40	NH_2-TTTTTTTTTTTTTTTTTTTTTTTTTTTTTTTTTTTTTTTT
R40	COOH-AAAAAAAAAAAAAAAAAAAAAAAAAAAAAAAAAAAAAAAA
F50	NH_2-TTTTTTTTTTTTTTTTTTTTTTTTTTTTTTTTTTTTTTTTTTTTTTTTTT
R50	COOH-<br>AAAAAAAAAAAAAAAAAAAAAAAAAAAAAAAAAAAAAAAAAAAAA<br>AAAAA
F60	NH_2-<br>TTTTTTTTTTTTTTTTTTTTTTTTTTTTTTTTTTTTTTTTTTTTTTTTTTTTTT<br>TTTTTT
R60	COOH-<br>AAAAAAAAAAAAAAAAAAAAAAAAAAAAAAAAAAAAAAAAAAAAA<br>AAAAA AAAAAAAAAA
F70	NH_2-<br>TTTTTTTTTTTTTTTTTTTTTTTTTTTTTTTTTTTTTTTTTTTTTTTTTTTTTT<br>TTTTTTTTTTTTTTTT
R70	COOH-<br>AAAAAAAAAAAAAAAAAAAAAAAAAAAAAAAAAAAAAAAAAAAAA<br>AAAAA AAAAAAAAAAAAAAAAAAAA
S1 for Ng	TCCTGGTCTCCGCTCCCCTCTGAGCCCCGTTAAACCCAAAGAATGTCTG<br>AGGGGAGCCACCCTCGGGGCCCAGGCCCCAGAGTCACACAT

S2 for Ng	FAM-ATGTGTGACTCTGGGGCCTGGGCCCCGAGGGTGGCTCCCCTCAGACATTCTTTGGGTTTAACGGGGCTCAGAGGGGAGCGGAGACCAGGA
S1 for dcas9	TTCAGAGGAACCAGGGACCTCGGGCACCCCAGAGCCCCTCGGGCCCGCCTCCAGGCGCCCTCCTGGTCTCCGCTCCCCTCTGAGCCCCGTTAAACCCAAA
S2 for dCas9	FAM-TTTGGGTTTAACGGGGCTCAGAGGGGAGCGGAGACCAGGAGGGCGCCTGGAGGCGGGCCCGAGGGGCTCTGGGGTGCCCGAGGTCCCTGGTTCCTCTGAA
g1	P-CCCGCCTCCAGGCGCCCTCCTGGT
g2	P-CAGAGAGAGGGGCAGAACCAACCC
g3	P-TAAACCCAAAGAATGTCTGAGGGG
g4	P-AGACATTCTTTGGGTTTAACGGGG
gDNA3 linker	SH-TTTTTTAAACCCAAAGAATGTCTGAGGGG
T13-1	COOH-AAAAAAAAAAACACTACGTCAGAACAGCTTGCATCACTGGTCACCAGAGTA
T13-2	NH_2-ACGAGCGAGTTGATGTGATGCAAGCTGAATGCGAGGGTCCT
T13-3	NH_2-TCAACTCGCTCGTAACTACACTGTGCAATACTCTGGTGACC
T13-4	NH_2-TCTGACGTAGTGTATGCACAGTGTAGTAAGGACCCTCGCAT
T17-2	NH_2-TATCACCAGGCAGTTGACAGTGTAGCAAGCTGTAATAGATGCGAGGGTCCA ATAC

T17-3	NH_2- TCAACTGCCTGGTGATAAAACGACACTACGTGGGAATCTACTATGGCGG CT CTTC
T17-4	NH_2- TTCAGACTTAGGAATGTGCTTCCCACGTAGTGTCGTTTGTATTGGACCCT C GCAT
T17-1	COOH- AAAAAAAAAAACATTCCTAAGTCTGAAACATTACAGCTTGCTACACGA GAAGAGCCGCCATAGTA
T26-1	COOH- AAAAAAAAAAGCCTGGAGATACATGCACATTACGGCTTTCCCTATTAGA AGGTCTCAGGTGCGCGTTTCGGTAAGTAGACGGGACCAGTTCGCC
T26-2	NH_2- CGCGCACCTGAGACCTTCTAATAGGGTTTGCGACAGTCGTTCAACTAGA ATGCCCTTTGGGCTGTTCCGGGTGTGGCTCGTCGG
T26-3	NH_2- GGCCGAGGACTCCTGCTCCGCTGCGGTTTGGCGAACTGGTCCCGTCTA CTTACCGTTTCCGACGAGCCACACCCGGAACAGCCC
T26-4	NH_2- GCCGTAATGTGCATGTATCTCCAGGCTTTCCGCAGCGGAGCAGGAGTCC TCGGCCTTTGGGCATTCTAGTTGAACGACTGTCGC
RNA-168	AGGACAGCCG TTGCCCTAGT GGTTTCGGAC ACACCGCCAA CGCTCAGTGC GGTGCTACCG ACCCGAGGTC AAGTCCCGGG GGAGGAGAAG AGAGGCTTCC CGCCTAGAGC ATTTGCAAGT CAGGATTCTC TAATCCCTCT GGGAGAAGGG TATTCGGCTT GTCCGCTA
RNA-351	CCAATGGGCG CGGGTCCCCC TAGCCCCGGC GGGGATCCGG ATGGGGACGA TGGCGGAAAC AACTCCCAAT ATCCATCTGC TTCTGGCTCT TCTGGGAACA CCCCCACCCC ACCGAACGAT GAGGAACGTG AATCTAATGA AGAGCCCCCA CCGCCTTATG

AGGACCTAGA TTGGGGCAAT GGCGACCGTC ACTCGGACTA
TCAACCACTA GGAAACCAAG ATCCAAGTTT GTACTTGGGA
TTGCAACACG ACGGGAATGA CGGGCTCCCT CCCCCTCCCT
ACTCTCCACG GGATGACTCA TCTCAACACA TATACGAAGA
AGCGGGCAGA GGAAGTATGA ATCCAGTATG C

gDNA for RNA-168	P-AAATGCTCTAGGCGGGAAGCCTCT
gDNA for RNA-351	P-CCAATCTAGGTCCTCATAAGGCGG
RT-PCR F1 for 168	TACCCTTCTCCCAGAGGG
RT-PCR R1 for 168	CAGTGCGGTGCTACCGAC
RT-PCR F2 for 168	CCCTTCTCCCAGAGGGATTA
RT-PCR R2 for 168	GGTTTCGGACACACCGCCAA
RT-PCR F3 for 168	CCCTTCTCCCAGAGGGAT
RT-PCR R3 for 168	TTTCGGACACACCGCCAA
RT-PCR F1 for 351	AGTAGGGAGGGGGAGGGA
RT-PCR R1 for 351	GGGACGATGGCGGAAACA
RT-PCR F2 for 351	AGAGTAGGGAGGGGGAGGGA
RT-PCR R2 for 351	GATGGGGACGATGGCGGAAA

RT-PCR F3 for 351	GGAGAGTAGGGAGGGGGAGGGA
RT-PCR R3 for 351	GATGGGGACGATGGCGGAAACA
PCR F for EBV plus	GTGTGACTCTGGGGCCTGGGCCCC
PCR R for EBV plus	CCCTCCTTTTGTAAACAATGCATT
PCR F for EBV	GGTTCTGCCCCTCTCTCTGTCC
PCR R for EBV	AGGGGAGCGGAGACCAGGAG
PCR F for SPR	biotin-GTGTGACTCTGGGGCCTGGGCCCC
PCR R for SPR	biotin-CCCTCCTTTTGTAAACAATGCATT
EBV plus	TTCCCTCCTTTTGTAAACAATGCATTCATCGACCTTCCAGCCGGGGTTGG TTCTGCCCCTCTCTCTGTCCTTCAGAGGAACCAGGGACCTCGGGCACCC CAGAGCCCCTCGGGCCCGCCTCCAGGCGCCCTCCTGGTCTCCGCTCCCC TCTGAGCCCCGTTAAACCCAAAGAATGTCTGAGGGGAGCCACCCTCGG GGCCCAGGCCCCAGAGTCACACATCCGACACAACAACAGCATTCTCCT
F for SA	ATTTTTTTCGTAAATGCACTTG
R for SA	CGGTTAAATTAATGTACAAAGG
F for PA	GCAACACTTCCTTCTCCC
R for PA	CATAGAGTCGGTCCTGCC
gDNA for SA	P-CTTTGTTTCAGGTGTATCAACCAA
gDNA for PA	P-TGACCGGATGTTCGAAGGCCAGTC

Supplementary Table 2. Comparison of representative amplification-free strategy.

Method	Target	Sensitive	Stability	Real-time (Y/N)	Detection platform	Ref.
Multiple Cas13a	SARS-CoV-2 RNA	100 copies/μL	Unknown	Y	Portable mobile device	(2)
Cascade CRISPR nucleases of Cas13 and Csm6	SARS-CoV-2 RNA	30 copies/μL	Unknown	N	Centrifugal tube	(3)
Graphene field effect transistor	S protein and N protein of SARS-CoV-2	1.28 PFU/mL for S protein;1.45 PFU/mL for N protein	Unknown	N	Field effect transistor	(4)
Engineered LwaCas13a	SARS-CoV-2 RNA	~aM	Unknown	N	Centrifugal tube	(5)
Strand	SARS-	400 copies/μL	Unknown	Y	Portable smart	(6)

displacement reaction	CoV-2 variants, Alpha, Beta, Gamma				phone-based device	
Liquid-gated graphene field-effect transistor	SARS-CoV-2 RNA	1-2 copies/100μL	15 days in full serum	Y	Field effect transistor	(7)
Combined Cas13 and Cas12 approach	SARS-CoV-2 variants, Delta, Omicron	100 copies/μL	Unknown	Y	Fluidigm instrumentation	(8)
Cas13a-Graphene field-effect transistors	SARS-CoV-2 RNA	1 aM	Unknown	Y	Field effect transistor	(9)
Enzyme-labeled reported	SARS-CoV-2 RNA	~10 fM	Unknown	N	Naked-eye visualization	(10)
Wearable	Oestradiol	0.14 pM	56 days	Y	Wearable	(11)

aptamer nano-biosensor					epidermis electrochemical patch	
TDN-Ng Wearable	EBV-related cfDNA and RNA of sepsis	0.3 fM	20 days in vitro; 17 days in vivo	Y	Fully integrated wearable	This work

Supplementary Table 3. The comparison of state-of-the-art MN patches for diagnostics

Strategy	Target	Sensitive	Stability	Continuous real-time (Y/N)	Integration (Y/N)	Ref.
Fully integrated enzyme-based MNs	Glucose, lactate, alcohol	0.32, 0.15, 0.5 mM for glucose, lactate, alcohol, respectively	Over 12 hours	Y	Y	(12)
Hyaluronic acid-based MNs for capturing target	Keratinocytes-derived chemokine, MCP-1, IFN-β, and IL-6	In the range of 321 fM to 1400 fM	Unknown	N	N	(13)
Ion-selective membrane based MNs	Ca^{2+}, K^+, Na^+ ions	0.01, 1, 10 mM for Ca^{2+}, K^+, Na^+ ions, respectively	5 days	Y	Y	(14)
Bilateral core-shell microneedle	Lactate	2 mM	20 days	N	N	(15)
MN-based electrochemical aptamer biosensing patch	Tobramycin, vancomycin	10 μM for tobramycin	1000 scans; 15 hours	Y	N	(16)
TDN-Ng MN	EBV-related cfDNA, RNA for sepsis	0.3 fM	14 days in vivo	Y	Y	This work

Supplementary Reference

(1) Angewandte Chemie International Edition, 2015, 54(7): 2151-2155.

(2) Cell, 2021, 184(2): 323-333. e9.

(3) Nature Chemical Biology, 2021, 17(9): 982-988.

(4) Proceedings of the National Academy of Sciences, 2022, 119(28): e2206521119.

(5) Nature Chemical Biology, 2023, 19(1): 45-54.

(6) Nature Biomedical Engineering, 2022, 6(8): 957-967.

(7) Nature Biomedical Engineering, 2022, 6(3): 276-285.

(8) Nature Medicine, 2022, 28(5): 1083-1094.

(9) Angewandte Chemie, 2022, 134(32): e202203826.

(10) Journal of the American Chemical Society, 2022, 144(36): 16310-16315.

(11) Nature Nanotechnology, 2023, https://doi.org/10.1038/s41565-023-01513-0.

(12) Nature Biomedical Engineering, 2022, 6(11): 1214-1224.

(13) Advanced Functional Materials, 2023, 2301659, https://doi.org/10.1002/adfm.202301659.

(14) Microsystems & Nanoengineering, 2023, 9(1): 25.

(15) Chemical Engineering Journal, 2023, 455: 140730.

(16) Science Advances, 2022, 8(38): eabq4539.
