# Supplementary material for: Long-term monitoring of ultratrace nucleic acids using tetrahedral nanostructure-based NgAgo on wearable microneedles
Source: Nat Commun. 2024 Mar 2;15:1936. doi: 10.1038/s41467-024-46215-w (PMC10908814; doi:10.1038/s41467-024-46215-w)
Supplement: Supplementary file 3 — Description of Additional Supplementary Files [file 41467_2024_46215_MOESM3_ESM.pdf]

<u>Description of Additional Supplementary Files</u>

Supplementary Movie Legends

Supplementary Movie 1: Molecular dynamic simulation of Ng protein and gDNA

Supplementary Movie 2: Stretchable TPU patch under different distortion

Supplementary Movie 3: Demonstration of integrated wearable

Supplementary Movie 4: Demonstration of integrated electronics on immunodeficiency mouse
