# Supplementary material for: Long-term monitoring of ultratrace nucleic acids using tetrahedral nanostructure-based NgAgo on wearable microneedles
Source: Nat Commun. 2024 Mar 2;15:1936. doi: 10.1038/s41467-024-46215-w (PMC10908814; doi:10.1038/s41467-024-46215-w)
Supplement: Supplementary file 8 — Reporting Summary [file 41467_2024_46215_MOESM8_ESM.pdf]

nature portfolio

Corresponding author(s): Xueen Fang

Last updated by author(s): Feb 8, 2024

Reporting Summary

Nature Portfolio wishes to improve the reproducibility of the work that we publish. This form provides structure for consistency and transparency in reporting. For further information on Nature Portfolio policies, see our Editorial Policies and the Editorial Policy Checklist.

Statistics

For all statistical analyses, confirm that the following items are present in the figure legend, table legend, main text, or Methods section.

n/a	Confirmed	
☐	☒	The exact sample size (*n*) for each experimental group/condition, given as a discrete number and unit of measurement
☐	☒	A statement on whether measurements were taken from distinct samples or whether the same sample was measured repeatedly
☐	☒	The statistical test(s) used AND whether they are one- or two-sided<br>*Only common tests should be described solely by name; describe more complex techniques in the Methods section.*
☒	☐	A description of all covariates tested
☒	☐	A description of any assumptions or corrections, such as tests of normality and adjustment for multiple comparisons
☐	☒	A full description of the statistical parameters including central tendency (e.g. means) or other basic estimates (e.g. regression coefficient) AND variation (e.g. standard deviation) or associated estimates of uncertainty (e.g. confidence intervals)
☐	☒	For null hypothesis testing, the test statistic (e.g. *F*, *t*, *r*) with confidence intervals, effect sizes, degrees of freedom and *P* value noted<br>*Give P values as exact values whenever suitable.*
☒	☐	For Bayesian analysis, information on the choice of priors and Markov chain Monte Carlo settings
☒	☐	For hierarchical and complex designs, identification of the appropriate level for tests and full reporting of outcomes
☒	☐	Estimates of effect sizes (e.g. Cohen's *d*, Pearson's *r*), indicating how they were calculated

Our web collection on statistics for biologists contains articles on many of the points above.

Software and code

Policy information about availability of computer code

Data collection	The page gel and agarose gel electrophoresis was imaged by EPS 300 and 4100 digital gel image system (Tannon Corp.). All the mechanical testing of the wearable patch and microneedle patch was performed on Instron 5966 electronic universal testing machine (Instron, USA). PCR was performed on a fluorescent quantitative PCR detection system (LineGene 9660, Hangzhou Bioer Technology Co., Ltd., Hangzhou, China). All the electrochemical characterization, acquisition, and testing was performed with a electrochemical workstation CHI 1030 and autolab (Nova 1.7). SEM was conducted on Zeiss Gemini SEM500 FESEM and VEGA 3 XMU (TESCAN Co., Czech). Animal bioimaging was performed with in Vivo Xtreme (Bruker, USA). Finite element analysis of the wearable patch was collected by COMSOL Multiphysics 5.3.
Data analysis	All the electrochemical data was analyzed by Origin software (version 2018) and Zview software (version 3.1). Microsoft Excel (version 2016)was used to analyze mechanical testing data . Primer premiere 5.0 (version 5.0), Generunner software (version 6.5), and NCBI database was used for PCR experiments. The next-generation sequencing was analyzed by Chromas (version 2.3). The images of PAGE gel results were analyzed by Image J (version 1.51k). All the animal bioimaging results were analyzed by Bruke MI SE (version 7.2).

For manuscripts utilizing custom algorithms or software that are central to the research but not yet described in published literature, software must be made available to editors and reviewers. We strongly encourage code deposition in a community repository (e.g. GitHub). See the Nature Portfolio guidelines for submitting code & software for further information.

Data

Policy information about availability of data

All manuscripts must include a data availability statement. This statement should provide the following information, where applicable:

- Accession codes, unique identifiers, or web links for publicly available datasets
- A description of any restrictions on data availability
- For clinical datasets or third party data, please ensure that the statement adheres to our policy

All data supporting the findings of this study are available within the article and its supplementary files. Any additional requests for information can be directed to, and will be fulfilled by, the corresponding authors. Source data are provided with this paper. The GenBank data generated in this study have been deposited in the NCBI database under accession code No. A10072.1, No. GU205107.1, No. M87778.1, MZ067393.1, D30812.1 for EBV BamHI-W, EBV EBER-2, EBV LMP 2A, SA strain, PA strain respectively (accessible link, https://www.ncbi.nlm.nih.gov/).

Research involving human participants, their data, or biological material

Policy information about studies with human participants or human data. See also policy information about sex, gender (identity/presentation), and sexual orientation and race, ethnicity and racism.

Reporting on sex and gender	One male and one Female volunteers were enrolled in this study. We have reported gender information on the enrolled volunteers, which is determined by self-reporting.
Reporting on race, ethnicity, or other socially relevant groupings	All the enrolled volunteers were not categoried by race, ethnicity, or other socially relevant groupings, and these factors are not relevant to this study.
Population characteristics	Healthy Subject (female and male, 23-32 years).
Recruitment	Samples were randomly recruited from our research group members in the age of 23-32 years old (one female participant and one male participant). They are with different self-reported activity level, and we didn't know their genetic disease.
Ethics oversight	The human Ethics Committee of Fudan University, China. The statement of informed consent obtained from the participants was listed in the paper.

Note that full information on the approval of the study protocol must also be provided in the manuscript.

Field-specific reporting

Please select the one below that is the best fit for your research. If you are not sure, read the appropriate sections before making your selection.

☒ Life sciences ☐ Behavioural & social sciences ☐ Ecological, evolutionary & environmental sciences

For a reference copy of the document with all sections, see nature.com/documents/nr-reporting-summary-flat.pdf

Life sciences study design

All studies must disclose on these points even when the disclosure is negative.

Sample size	For animal experiment, the 4-week-old female Balb/c nude mice (n=3 for each group) were purchased from Beijing Vital River Laboratory Animal Technology Co., Ltd. (Beijing, China), whose sample sizes were sufficient for two-way ANOVA analysis in the study.
Data exclusions	No data were excluded
Replication	Data acquisition was conducted on different groups (n=3 for each group) for one experimental performance to verify the feasibility. And three experimental performances were conducted in different period of time. The time interval of each experimental performance was 1 month.
Randomization	The BALB/c nude mice with different activity levels were randomly allocated into the groups.
Blinding	No blinding measures were taken deliberately for Balb/c nude mice. Because those Balb/c nude mouse with target nucleic acid were used as positive models and healthy Balb/c nude mouse were used as negative models, which provided practical models for the following experiments. All the animal experiment data was processed together by multiple research group members.

Reporting for specific materials, systems and methods

We require information from authors about some types of materials, experimental systems and methods used in many studies. Here, indicate whether each material, system or method listed is relevant to your study. If you are not sure if a list item applies to your research, read the appropriate section before selecting a response.

Materials & experimental systems

n/a	Involved in the study
☒	☐ Antibodies
☐	☒ Eukaryotic cell lines
☒	☐ Palaeontology and archaeology
☐	☒ Animals and other organisms
☒	☐ Clinical data
☒	☐ Dual use research of concern
☒	☐ Plants

Methods

n/a	Involved in the study
☒	☐ ChIP-seq
☒	☐ Flow cytometry
☒	☐ MRI-based neuroimaging

Eukaryotic cell lines

Policy information about cell lines and Sex and Gender in Research

Cell line source(s)	CNE cells line was purchased from Beina Chuanglian Biology Research Institute (Catalog NO. BNCC341794, Beijing, China). Hela-GFP-Luc cells line was purchased from Quicell corporation (Catalog NO. QuiCell-H548, Shanghai, China).
Authentication	All the cell lines were not authenticated by the authors. And all the cell lines were authenticated by cells supplier incorporations.
Mycoplasma contamination	Mycoplasma tests were conducted and the results were negative.
Commonly misidentified lines (See ICLAC register)	No commonly misidentified cell lines were used

Animals and other research organisms

Policy information about studies involving animals; ARRIVE guidelines recommended for reporting animal research, and Sex and Gender in Research

Laboratory animals	4-week-old female Balb/c nude mice were in the same housing condition, which was in the cycle of 6-hour dark/18-hour light (23 °C ±2°C, 30%-40% of ambient humidity).
Wild animals	The study did not involve wild animals.
Reporting on sex	Female mice were used in this study. The findings didn't apply to only one sex. Sex was not considered in study design. The data disaggregated for sex has not been collected for sex-based analysis, because sex was not relevant to the experiments.
Field-collected samples	The study did not involve the collected samples from field.
Ethics oversight	Animals were cared for and maintained under the Guidelines of Laboratory Animals of Fudan University and approved by the Animal Ethics Committee of Fudan University, China.

Note that full information on the approval of the study protocol must also be provided in the manuscript.

Plants

Seed stocks	*Report on the source of all seed stocks or other plant material used. If applicable, state the seed stock centre and catalogue number. If plant specimens were collected from the field, describe the collection location, date and sampling procedures.*
Novel plant genotypes	*Describe the methods by which all novel plant genotypes were produced. This includes those generated by transgenic approaches, gene editing, chemical/radiation-based mutagenesis and hybridization. For transgenic lines, describe the transformation method, the number of independent lines analyzed and the generation upon which experiments were performed. For gene-edited lines, describe the editor used, the endogenous sequence targeted for editing, the targeting guide RNA sequence (if applicable) and how the editor was applied.*
Authentication	*Describe any authentication procedures for each seed stock used or novel genotype generated. Describe any experiments used to assess the effect of a mutation and, where applicable, how potential secondary effects (e.g. second site T-DNA insertions, mosiacism, off-target gene editing) were examined.*
